# Role of ICAM1 in tumor immunity and prognosis of triple-negative breast cancer

**DOI:** 10.3389/fimmu.2023.1176647

**Published:** 2023-08-21

**Authors:** Qin Zhou, Jiawei Xu, Yan Xu, Shaokun Sun, Jian Chen

**Affiliations:** ^1^ Suzhou Medical College of Soochow University, Suzhou, China; ^2^ Department of Breast surgery, Affiliated Kunshan Hospital of Jiangsu University, Kunshan, China

**Keywords:** triple-negative breast cancer, metastasis, ICAM1, prognosis, immunotherapy

## Abstract

**Background:**

Treating triple-negative breast cancer (TNBC) is a difficult landscape owing to its short survival times and high risk of metastasis and recurrence among patients. Although involved in tumor invasion and metastasis, the mechanism of action of intercellular adhesion molecule 1 (ICAM1), a trans-membrane glycoprotein, in TNBC is ambiguous.

**Methods:**

We examined ICAM1’s role in TNBC, focusing on its expression, cell survival, mutation, and tumor immunity. Then, a risk score model was created utilizing co-expressed genes associated with ICAM1. According to their respective risk scores, we divided patients into high- and low-risk groups. Immune function, drug susceptibility differences, and somatic variants were analyzed in the high-and low-risk groups. And we used the CMap database to predict potential medications. Then, TNBC cells with low expression of ICAM-1 were co-cultured with PMA-treated THP-1 cells and CD8 T cells. In addition, We detected the expression of PD-1 and CTLA4 of low ICAM-1 expressing TNBC cells when they were cocultured with CD8 T cells.

**Results:**

ICAM1 was found to be involved in leukocyte cell adhesion, motility, and immune activation. Patients with low-ICAM1 group had shorter disease-free survival (DFS) than those with high-ICAM1 group. The group with elevated levels of ICAM1 exhibited significantly increased levels of T-cell regulation, quiescence in natural killer (NK) cells, and M1 macrophage. ICAM1 expression was correlated with immune checkpoint drugs. The prognostic ability of the risk score model was found to be superior to that of individual genes. Patients categorized as high-risk exhibited elevated clinical stages, showed higher M1 macrophage numbers, and were able to benefit better from immunotherapy. Individuals belonging to the high-risk group exhibit significantly elevated mutation rates in TP53, TTN, and SYNE1 genes, along with increased TMB and PD-L1 levels and decreased TIDE scores. These findings suggest that immunotherapy may be advantageous for the high-risk group. Furthermore, low expression of ICAM1 was found to promote polarization to M2 macrophages along with T-cell exhaustion.

**Conclusion:**

In conclusion, Low ICAM1 expression may be related to immune escape, leading to poor treatment response and a worse prognosis.

## Introduction

Breast cancer is the primary mortality cause in the female population. with more than 2.2 million affected globally (1). By 2050, more than 3 million breast cancer cases are predicted to be diagnosed annually across the world (2). The earliest classification of breast cancer was based on clinicopathological features, such as *in situ* carcinoma and metastases (3). Breast cancer updated categorization has resulted in recognition of four distinct sub types, involving triple-negative breast cancer (TNBC), human epidermal growth factor receptor (HER2)-over-expressing, luminal A, as well as luminal B. TNBC, which lacks the expression of the estrogen receptor (ER), progesterone receptor (PR), and human epidermal growth factor receptor 2 (HER2), makes up roughly 10–15% of breast cancer incidents (4). TNBC is characterized by early metastases, aggressive proliferation, poor prognosis, and survival (5). A challenging treatment landscape enhances the requirement for a more thorough comprehension of the molecular underpinnings behind the emergence as well as the progress of TNBC, which can help further identify suitable diagnostic markers and treatment targets.

Cell adhesion molecules (CAMs) are primarily found on endothelial and immune cells’ surfaces and contribute significantly to the ability of lymphocytes to bind to target cells. The trans-membrane glycoprotein receptor ICAM1, which belongs to the immunoglobulin (Ig) family, was identified first in mice (6). ICAM1 in association with macrophage 1 antigen (MAC-1), CD11b/CD18, lymphocyte function-associated antigen (LFA)-1, CD11a/CD18, and CD11b/CD18 promotes tumor metastasis by activating the immune system and enhancing cell signaling and inflammatory responses (7, 8). The expression of ICAM1 is observed to be low in vascular endothelial cells as well as specific lymphocytes. However, in the presence of inflammatory cytokines involving interleukin (IL)-1 along with tumor necrosis factor (TNF)-α, ICAM1 expression increases, improving adhesion between endothelial cells and leukocytes, leading to inflammation (9). On one hand, soluble ICAM1 released from the cell surface causes local immunosuppression in patients with gastric cancer (10). On the other hand, ICAM1 may have a role in tumor metastasis (7). The emergence of gastric cancer is linked to ICAM1 levels, and hence ICAM1 can potentially be a biomarker for early diagnosis and prognosis of gastric cancer (11). Other molecules, such as microRNAs (miRNAs), also regulate ICAM1 levels. MiR-335-5p binds to the ICAM1 3′UTR (untranslated region) and inhibits ICAM1 expression, thereby limiting the migration, invasion, and metastasis of thyroid cancer cells (12). ICAM1 is reported to involve different cancer kinds, such as ovarian cancer (13), colorectal (14), renal cell carcinoma (15), and prostate (16). ICAM1 expression levels are higher in the TNBC subtype than in other breast cancer types (17). Moreover, ICAM1 increases apoptosis resistance *via* transforming growth factor-beta (TGF-β)/SMAD signaling pathway, leading to bone metastases in TNBC (18). However, the role of ICAM1 in TNBC is not yet clear.

The present investigation assessed ICAM1 expression levels association with TNBC patients’ prognosis. The participants were split into two groups, specifically high- and low-ICAM1 groups, on the basis of median ICAM1 expression. Differential genes between groups and the related pathways, gene mutation, and protein interaction network were analyzed. We used to gene-weight co-expression analysis on TNBC samples to identify ICAM1 co-expressed genes and analyze their functions and pathways. A risk score model was developed utilizing the co-expression genes of ICAM1. The model was utilized to categorize tissue samples obtained from patients into high- and low-risk groups. Immune infiltration along with tumor microenvironment among high- and low-risk groups was compared. Small molecules’ therapeutic effects were then analyzed using the Connectivity Map (CMap) database in patients with TNBC. TNBC cells with low ICAM1 expression subjected to co-culture with THP-1 cells underwent treatment with phorbol 12-myristate 13-acetate (PMA) and CD8 T cells to study the alterations in ICAM1 expression in the tumor microenvironment.

## Methods

### Acquisition of datasets

The Cancer Genome Atlas (TCGA) database were employed to acquire survival, clinical characteristics, and gene expression data of breast cancer. To identify TNBC samples, we searched the database using estrogen receptor (ER), progesterone receptor (PR), as well as HER-2 keywords. Incomplete survival data and negative samples were disregarded. We can have calculated the expression level of each gene in all samples, filtered out low-expressing genes, and excluded poor-quality samples retaining only high-quality samples. We searched Gene Expression Omnibus (GEO) database for TNBC gene expression data to validate risk score model. ICAM1 mutations across 33 cancer types were analyzed using the cBioPortal for Cancer Genomics tool. We also used Human Protein Atlas (HPA) database to determine ICAM1 distribution at tissue and cellular levels.

### Prognostic analysis of ICAM1 in breast cancer

The TCGA dataset was utilized to gather prognostic data on breast cancer patients, including disease-free survival (DFS) and survival status. Firstly, breast cancer patients were grouped into 4 categories (luminal A, luminal B, TNBC, and HER-2 over-expressed) based on clinical data. According to median ICAM1 expression levels, individuals were split into high- and low-ICAM1 groups. R survival package survfit function was utilized to assess survival contrast among high- and low-ICAM1 groups, and ggsurvplot function was employed to create a survival curve and add annotations such as hazard ratio (HR) as well as p-value.

### Differential pathway analysis of high- and low-ICAM1 groups

The differential expression analysis (Log2FC) and multiple adjusted p values were calculated utilizing R limma package. The fold change values for every gene in the high- and low-ICAM1 groups were determined, as well as a differential analysis was conducted to identify differential genes. Genes that exhibited differential expression showed a p-value < 0.05 and a Log2FC absolute value greater than 1. In addition, the enrichGO function of the ClusterProfiler package was utilized to conduct Gene Ontology (GO) enrichment analysis on differentially expressed genes. This analysis encompasses three levels of biological processes, cellular components, as well as molecular activities. Furthermore, we conducted Kyoto Encyclopedia of Genes and Genomes (KEGG) enrichment analysis utilizing Enrichr (https://maayanlab.cloud/Enrichr/) and Gene Set Enrichment Analysis (GSEA), furtherly investigating differential genes.

### High- and low-ICAM1 groups immunological analysis

To accurately determine the composition and quantify the immune cells in patient samples in the TNBC tumor microenvironment, we used the CIBERSORT algorithm. The algorithm utilized the Leukocyte Signature Matrix file and Expression Data (LM22.txt) as input files. Statistical tests were carried out utilizing rstatix package. Wilcox rank sum test was applied to investigate immune cells ratio variations between the high- and low-ICAM1 groups, and results were displayed using the ggpubr R package.

### ICAM1 protein interaction network analysis

The GeneMANIA online database (https://genemania.org) employs a quick heuristic approach for predicting gene function across various functional association networks. In-depth genomic and proteomic data are used to identify functionally related genes, and each functional genomic dataset is weighted following the expected query value. GeneMANIA was utilized in this research to examine the network of interactions between the ICAM1 proteins. Proteins in the network were subjected to the KEGG annotation study using the Enrichr online tool, while proteins in the protein-protein interaction (PPI) network was analyzed for GO enrichment employing R clusterProfiler software.

### Weighted gene co-expression analysis

The R WGCNA package was utilized to construct gene co-expression networks in TNBC. We initially applied the goodSamplesGenes function to remove any missing genes and samples before clustering the samples and eliminating outliers from the dataset. The correlation coefficient needs to be calculated for the traditional analysis of the association between two genes, but this method is that a threshold needs to be artificially defined to confirm whether the gene expression is similar. WGCNA uses the idea of a soft-threshold to solve this problem well, and used the pickSoftThreshold function to to pick the best soft-threshold. Genes with comparable expression patterns should be grouped together into a single module using the blockwiseModules function for network creation and module discovery. After the correlation between each module and phenotype was computed, the modules that co-expressed the ICAM1 gene were chosen for further investigation, and a module-correlation heat map was created using the labelledHeatmap function. The GO and KEGG enrichment analyses were conducted on the co-expressed genes of ICAM1 to gain a deeper understanding of their biological significance.

### Risk score model construction

We conducted Cox regression analysis on 97 patients with TNBC according to genes intersecting in the blue module and PPI network, combined with their expression levels and survival statistics from tumor samples. Patients whose survival times were not reported were removed from the analysis. We used the R caret package to divide the patients into training and test sets at a 7:3 proportion. The training set was utilized for the training model, followed by an assessment of the test set to prevent the model from over-fitting, and model training process employed 10-fold cross-validation. We performed Lasso Cox analysis and only included genes exhibiting non-zero regression coefficients in the final prognostic model. Patients were categorized into two groups, high-risk and low-risk, utilizing the median risk score derived from the risk score model for every patient. The R Survival package was applied to generate a Kaplan-Meier survival curve, which investigates survival rate variations among high-risk as well as low-risk groups.

In GEO analysis, GSE58812 datasets, which contains 107 TN breast cancer patients and undertook robust functional annotation of the molecular entities, were chosen as the external validation set, and The model was employed to categorize patients into high- and low-risk groups, and subsequently, the risk score for every sample was determined. Furthermore, the study conducted survival difference analysis and utilized receiver operating characteristic curve (ROC) analysis confirming risk score model accuracy across the groups. Using the R timeROC function, we created a time-dependent ROC curve assessing risk score model predictive accuracy and estimated the area under the curve (AUC) for one, three, as well as five years. The prognostic significance of the risk score model has been verified through the utilization of both univariate and multivariate Cox regression analyses. A nomogram was created utilizing the R regression modeling strategies (rms) package to forecast the prospective survival rate of patients.

### Analysis of differences in immune function and drug susceptibility in high- and low-risk groups

Tumor purity variations, stromal score, and the immune score of the tumor immune microenvironment were estimated for both high-risk as well as low-risk groups. Scatter plots with risk scores were generated, and the correlation was examined using a linear fitting. R ESTIMATE package was utilized for immune cell quantification in high- as well as low-risk groups. The oncoPredict software was utilized to forecast the drug response and compare response variation to particular drugs among the high- as well as low-risk groups.

### Somatic variant analysis

TCGA dataset and R maftools package analysis were utilized to retrieve TNBC somatic mutation data. The oncoplot function was employed to create a waterfall chart of the top 20 gene mutations comparing high- as well as low-risk groups. Then, we analyzed mutation effects on ICAM1 protein structure using PyMOL v2.3.

### Compound treatment response prediction

We used the CMap database to predict potential medications. The up and down-regulated genes among high- as well as low-risk groups were fed into the database, along with the top 15, as well as the bottom 15 results, which were selected for a visualization based on median tau scores. The 3 dimensional (3D) structures of the drug candidates were visualized using the PubChem database.

### Cell culture

American Tissue Culture Collection (ATCC, Manassas, VA, USA) provided immortalized TNBC cell lines MDA-MB-231. These cells were cultured in RPMI1640 media with 10% fetal bovine serum (FBS). The MCF10A epithelial cell line was cultured in Dulbecco’s Modified Eagle’s media with 10% fetal bovine serum(FBS-DMEM). THP-1 cells (#GDC0100, China Center for Type Culture Collection, Wuhan, China) were cultured in RPMI1640 media with 10% fetal bovine serum (FBS). The cells were cultured and maintained under standard conditions of 37°C and humidity of 5% CO_2_.

### Quantitative real-time polymerase chain reaction

The Trizol reagent (Invitrogen, USA) was employed for the extraction of the total cellular RNA. High-quality RNA was quantified through UV analysis. Reverse transcription of isolated RNA into cDNA was carried out by employing the PrimeScript™ RT reagent kit (Takara Biomedical Technology, Beijing, China) with a gDNA eraser. The SYBR^®^ Premix Ex Taq™ was utilized to conduct real-time polymerase chain reaction (RT-PCR). (Takara Biomedical Technology, Beijing, China) on a LightCyclerR 480II real-time fluorescence quantitative PCR (qPCR) instrument (Roche, Shanghai, China). The 2^-ΔΔCT^ method was employed to calculate fold changes, while Glyceraldehyde 3-phosphate dehydrogenase (GAPDH) was utilized as a normalization agent for mRNA expression.

### Cell co-culture assays

Phorbol 12-myristate 13-acetate (PMA)-treated THP-1 cells (5 × 10^4^) or CD8 T cells were inoculated in transwell chambers. MDB-MA-231 cells with stable low expression of ICAM1 were inoculated in the lower transwell chamber. RPMI1640 medium containing 10% FBS was added for 48 h. Subsequently, Co-cultured PMA-treated THP-1 cells or CD8 T cells analysis were performed employing qPCR with enzyme-linked immunosorbent assay (ELISA), respectively.

### Cytokine analysis

After 24 h of co-culturing with MDA-MB-231 cells, the TNF-α, interferon (IFN)-γ, macrophage colony-stimulating factor (M-CSF), interleukin (IL)-1β, TGF-β1, chemokine (C-C motif) ligand 7 (CCL17) levels in the macrophage and CD8 T cells were examined using the ELISA kit (R&D systems, Bio-Techne, Shanghai, China) following recommendations provided by the manufacturer.

### *In vitro* cytotoxic assay

Cell-mediated cytotoxicity was assessed using a non-radioactive lactate dehydrogenase (LDH) release assay (Roche), in accordance with the manufacturer’s instructions. The measured values were adjusted by subtracting the spontaneous LDH release from both the target and effector cells. The percentage of specific lysis, indicating the extent of cell death, was calculated using the formula: (experimental release – target spontaneous release – effector spontaneous release) divided by (target maximum release – target spontaneous release). Each experiment was replicated three times, and the results were consistent.

### Statistical analyses

The data are shown in mean ± standard deviation (SD) format. Statistical analysis was conducted utilizing Statistical Package for Social Sciences (SPSS) version 22.0 (IBM Corp.) program. Group differences were assessed utilizing either a one-way ANOVA with Tukey’s *post hoc* test or a Student’s t-test. A statistically significant level was a p-value <0.05.

## Results

### ICAM1 gene expression analysis

**Figure 1:**
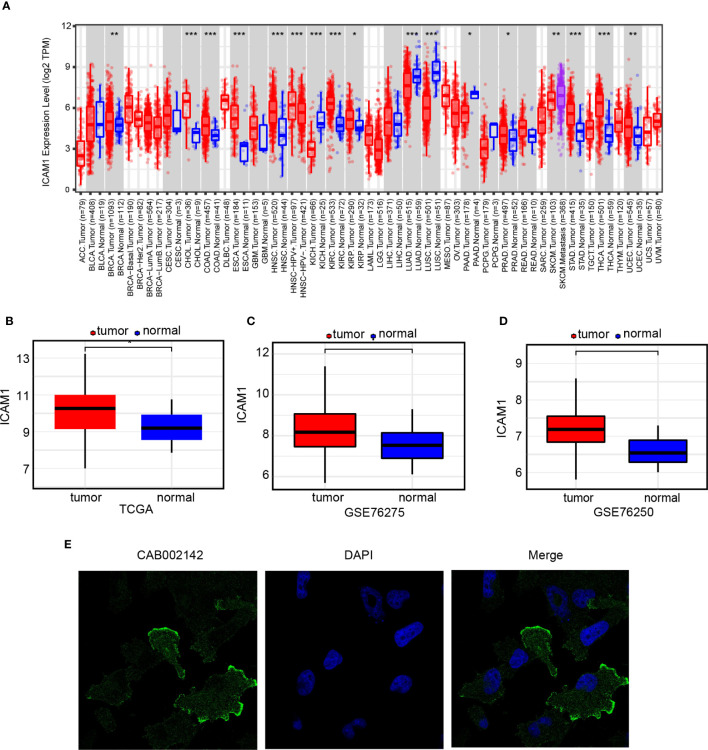
ICAM1 gene expression analysis. **(A)** ICAM1 expression levels in 33 common cancer types; **(B)** ICAM1 expression levels in the TNBC tumors and control tissues of the TCGA database; **(C)** ICAM1 in the tumors and control tissues of the GSE76275 dataset; **(D)** ICAM1 expression levels in the tumor and control tissues of the GSE76250 dataset; **(E)** HPA database analysis of ICAM1 subcellular localization. *p<0.05, **p<0.01, ***p<0.001.

ICAM1 expression pattern may vary in different types of cancer (**Figure 1A**). ICAM1 was weakly expressed in LUAD, SKCM, LUSC, as well as KICH but was strongly expressed in BRCA, CHOL, HNSC, KIRC, KIRP, STAD, THCA, as well as UCEC. TCGA, GSE76275, and GSE76250 datasets were chosen to evaluate ICAM1 expression in TNBC. Results demonstrated that ICAM1 expression was more abundant in TNBC than in paracancer tissues (**Figures 1B–D**), and ICAM1 is primarily localized to the plasma membrane and cytoplasm (**Figure 1E**).

### Analysis of clinical prognosis for ICAM1

**Figure 2:**
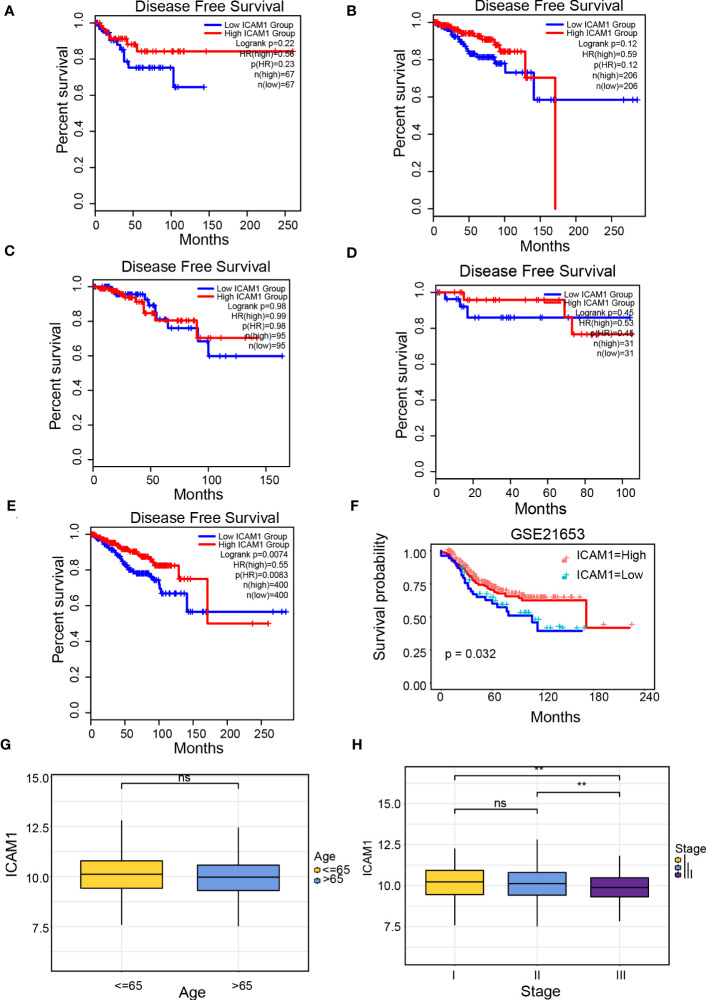
Clinical prognosis analysis of ICAM1. Survival curve of ICAM1 in: **(A)** TNBC; **(B)** luminal A type breast cancer; **(C)** luminal B type breast cancer; **(D)** HER-2 over-expressed type breast cancer; **(E)** all breast cancer types; **(F)** the GSE21653 datasets; **(G)** Correlation between ICAM1 expression level and age; **(H)** Correlation between ICAM1 expression level and clinical stage. **p<0.01, ns p>0.05.

Patients with breast cancer were categorized into 4 groups (luminal A, luminal B, TNBC, and HER-2 over-expressed), as well as the correlation between ICAM1 expression and DFS was analyzed. However, except for a trend toward better prognosis in the high-ICAM1 group, there was no association between ICAM1 and prognosis among the four groups (**Figures 2A–D**). We attempted to merge the data from the four types of breast cancer. Results identified that high-ICAM1 group patients had improved prognosis that was statistically significant (hazard ratio [HR]=0.55, p=0.0074; **Figure 2E**). The non-significant outcomes of the survival analysis were attributed to the small TNBC sample size. Hence, the GSE21653 dataset was analyzed, which revealed that patients belonging to the high-ICAM1 group exhibited a more favorable prognosis (**Figure 2F**). We then selected the clinical parameters of age and stage to examine the association between ICAM1 and TNBC. ICAM1 expression levels between patients aged 65 years and more than 65 years were found to be the same (**Figure 2G**). Furthermore, we found that stage I had the highest ICAM1 expression levels, followed by stage II, whereas stage III had the lowest (**Figure 2H**). As the clinical stage of TNBC increased, ICAM1 expression levels decreased. Therefore, we believe ICAM1 may act as a protective factor for TNBC.

### Pathway enrichment analysis of the high- and low-ICAM1 groups

**Figure 3:**
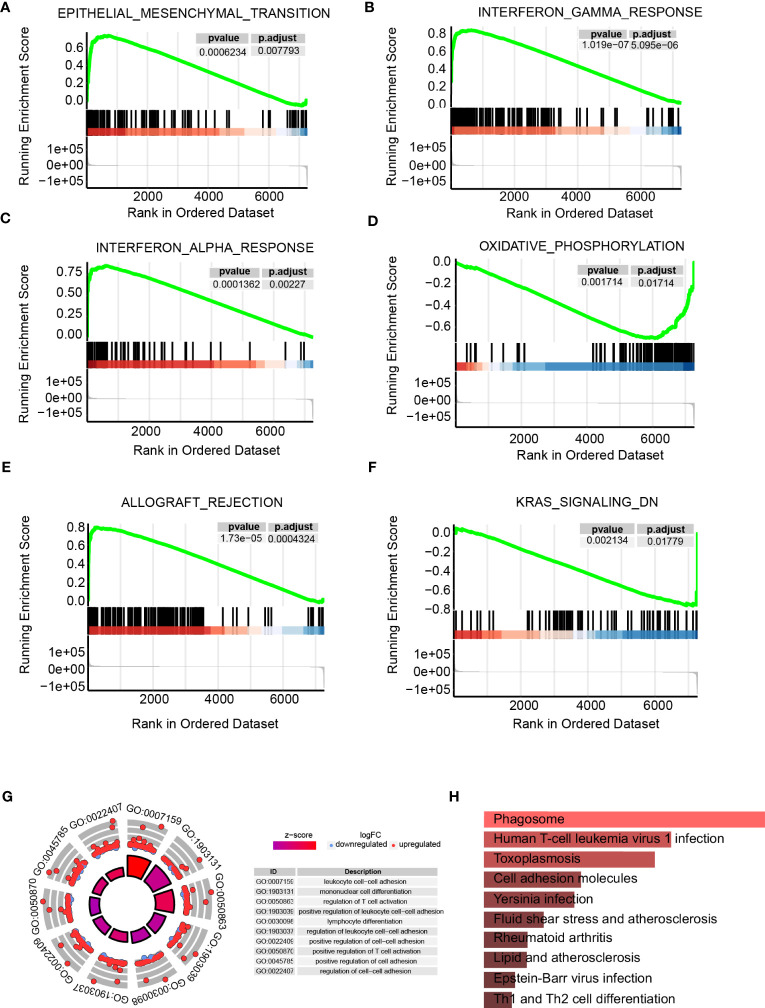
Pathway enrichment analysis of the high- and low-ICAM1 groups. **(A–F)** Gene set enrichment analysis; **(G)** GO enrichment analysis; **(H)** KEGG metabolic pathway enrichment analysis.

Individuals were categorized into high- as well as low-ICAM1 group on the basis of ICAM1 median expression. We established |log2FC|>2, p<0.05, and identified 8296 differential genes. The high-ICAM1 group had 3913 genes that were over-expressed and 4383 genes that were decreased among them (**Table S2**). GSEA of these differential genes showed that epithelial-mesenchymal transition, IFN response, and allograft rejection were over-expressed in the high-ICAM1 group (**Figures 3A–D**), whereas oxidative phosphorylation and KRAS signaling down pathway was decreased in the high-ICAM1 group (**Figures 3E, F**). The GO enrichment analysis revealed that differential genes were primarily involved in leukocyte cell adhesion, monocyte differentiation, control of T-cell activation, as well as leukocyte cell adhesion positive regulation (**Figure 3G**). KEGG enrichment analysis revealed that differential genes were included in pathways such as phagosomes, infection with human T-cell leukemia virus 1, *Toxoplasma gondii*, and cellular molecular adhesion (**Figure 3H**).

### ICAM1 mutation analysis

**Figure 4:**
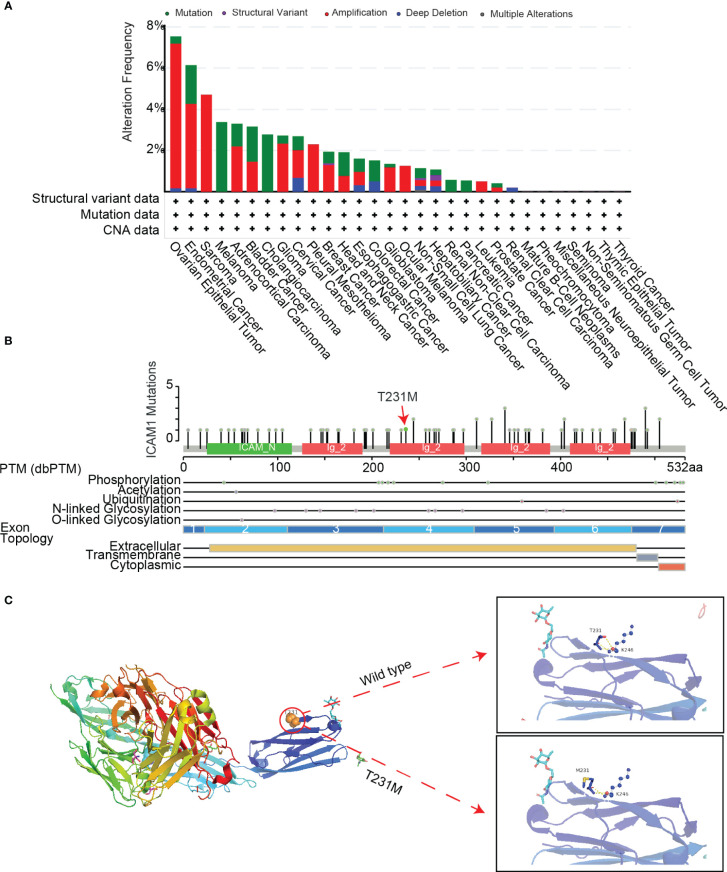
ICAM1 mutation analysis. **(A)** Mutation analysis of ICAM1 in common cancers; **(B)** Lollipop display of common mutations (point mutation and post-translational modification mutation) of ICAM1 on the gene structure; **(C)** Display of the secondary structure of ICAM1 protein and molecular interaction analysis after the T231M mutation.

To understand the role of common mutations in developing drug targets, we analyzed common ICAM1 mutation types in various cancers using cBioPortal datasets. Results showed that point mutations, structural variations, amplifications, deep deletions, as well as multiple modifications are the most common forms of ICAM1 mutations. An ICAM1 mutation is most often seen in ovarian epithelial tumors, whereas it is least frequently found in renal clear cell carcinoma. Further, some cancers, such as cholangiocarcinoma and leukemia, have only one point mutation in ICAM1. We also found that the frequency of ICAM1 mutation in breast cancer is about 2%, and the mutation is mainly in the form of amplification, accompanied by a point mutation and structural variation (**Figure 4A**). A lollipop diagram shows the location of mutations on the ICAM1 gene structure, with different color blocks indicating the different structural domains of the ICAM1. ICAM1 contains 4 immunoglobulin domains, and the T231M mutation frequency is the highest in breast cancer (red arrows). ICAM1 exhibits several post-translational modifications, including phosphorylation, acetylation, ubiquitination, N-linked glycosylation, and O-linked glycosylation, in addition to alterations at the transcriptional level (**Figure 4B**). Finally, we found that the side chains of T231 formed three hydrogen bonds with the backbone of K246 in the wild type, whereas a mutation of M231 weakened the hydrogen bonds between the side chain and K246 backbone (**Figure 4C**). Further, we screened the TNBC data from the cBioPortal database and found that copy number amplification was the type of ICAM1 variation, and there was no point mutation.

### ICAM1 protein interaction network

**Figure 5:**
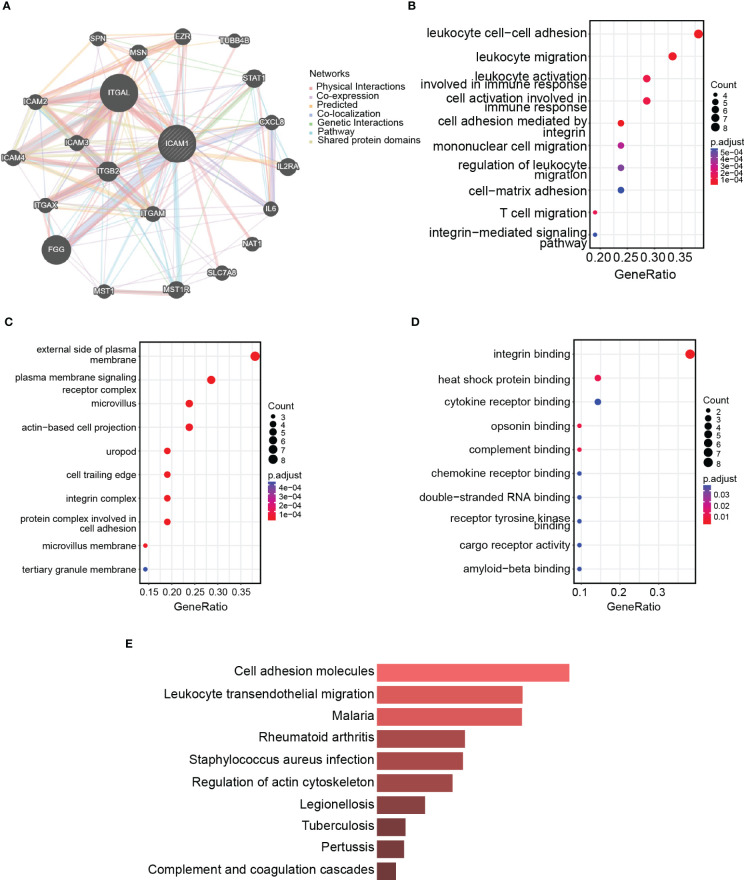
ICAM1 protein interaction network analysis. **(A)** PPI network for ICAM1; **(B)** Biological processes (BP) enrichment analysis; **(C)** Cellular components (CC) enrichment analysis; **(D)** Molecular function (MF) enrichment analysis; **(E)** KEGG enrichment analysis.

The ICAM1 protein interaction network consists of 20 nodes with 326 edges, and the interaction correlation is dominated by physical interactions (77.64%; **Figure 5A**). GO enrichment analysis findings demonstrated that interacting proteins are associated with processes, such as leukocyte migration, integrin-mediated cell adhesion, leukocyte cell adhesion, and leukocyte activation, implicated in immunological responses (**Figure 5B**). The interacting proteins are located on the plasma membrane exterior side, the plasma membrane signaling receptor complex, the microvillus, as well as the actin-based cell projection (**Figure 5C**). The interacting proteins are associated with molecular functions such as integrin binding, heat shock protein binding, cytokine receptor binding, opsonin binding, and complement binding (**Figure 5D**). The KEGG enrichment analysis revealed that interacting proteins participated in pathway such as *Staphylococcus aureus* infection, malarial pathogenesis, leukocyte transendothelial migration, and cell adhesion (**Figure 5E**). Therefore, ICAM1 is essential for leukocyte motility, intercellular adhesion of leukocytes, and leukocyte-associated immunological activation.

### Analysis of tumor infiltrates and tumor microenvironments in the high- and low-ICAM1 groups

**Figure 6:**
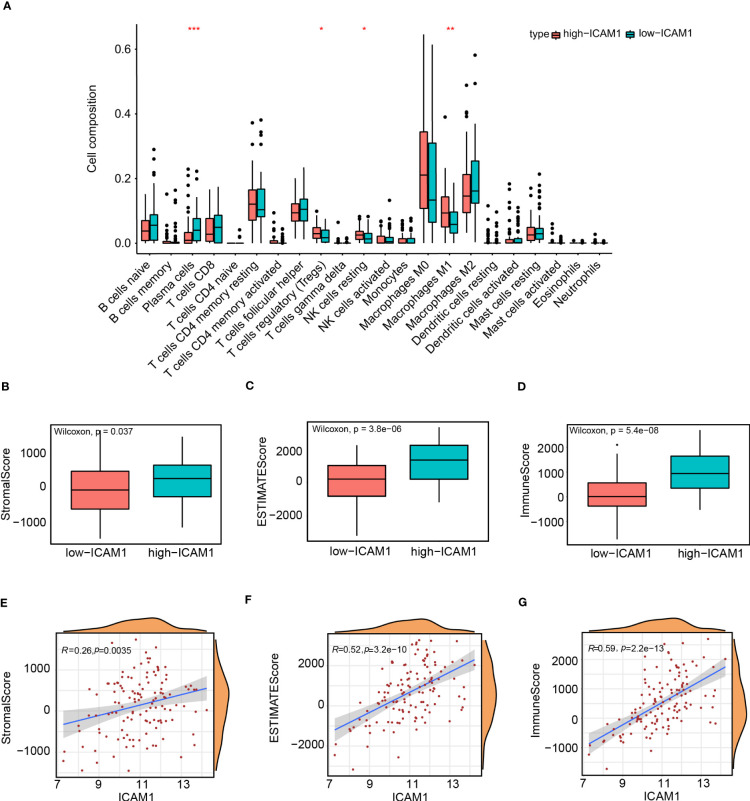
Analysis of tumor infiltrates and tumor microenvironment in the high- and low-ICAM1 groups. Correlation analysis between: **(A)** ICAM1 gene expression and immune infiltrating cells; **(B)** ICAM1 gene expression and stromal scores; **(C)** ICAM1 gene expression and estimate scores; **(D)** ICAM1 gene expression and immune scores; **(E)** ICAM1 gene expression and stromal scores; **(F)** ICAM1 gene expression and estimate scores; **(G)** ICAM1 gene expression with immune scores. *p<0.05, **p<0.01, ***p<0.001.

The immune infiltration cell variations among the high- and low-ICAM1 groups were examined using the CIBERSORT algorithm. The findings demonstrated that compared to the low-ICAM1 group, the high-ICAM1 group had considerably higher levels of T-cell regulation, natural killer (NK) cell quiescence, and M1 macrophage. Moreover, the low-ICAM1 group had higher plasma cells than the high-ICAM1 group (**Figure 6A**). The ESTIMATE algorithm was used for the analysis of stromal scores, estimate scores, as well as immune scores of the high- and low-ICAM1 groups. The high-ICAM1 group surpassed in terms of stromal cells (p=0.037, **Figure 6B**), tumor cell purity (p=3.8e-10, **Figure 6C**), and immune score (p=5.4e-08, **Figure 6D**). We examined ICAM1 expression levels’ association with stromal cells, tumor purity, and immunological scores. ICAM1 expression corresponded well with the stromal cell quantity (R-0.26, p=0.0035), tumor purity (R=0.52, p=3.2e-10), and immune score (R=0.59, p=2.2e-13, **Figures 6E–G**). Also, we found a favorable correlation between ICAM1 and six immunological checkpoint molecules (CD274, CTLA4, HAVCR2, LAG3, PDCD1, and PDCD1LG2; **Figure S1**).

### Weighted gene co-expression analysis

**Figure 7:**
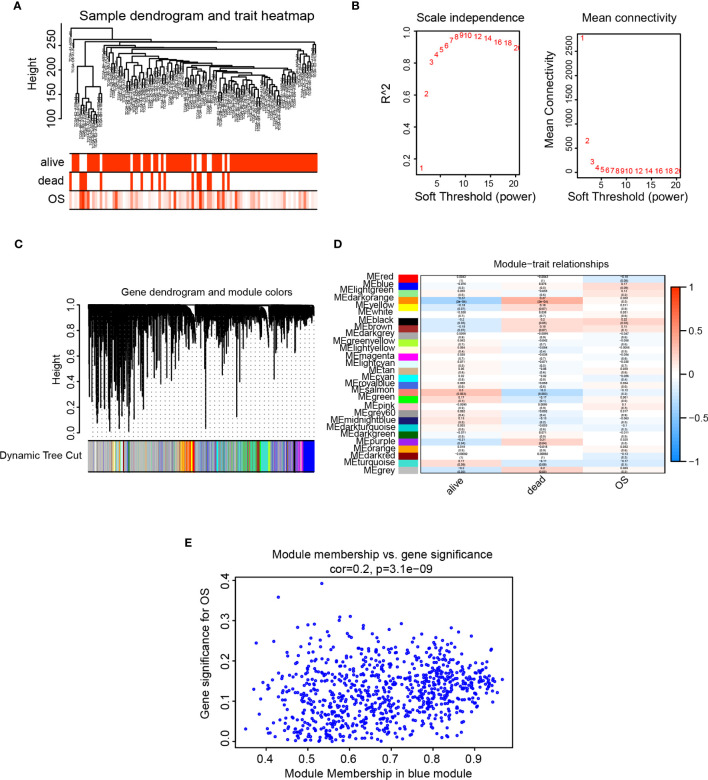
WGCNA analysis. **(A)** Analysis of sample level clustering combined with clinical traits; **(B)** Analysis of the scale-free fit index (left) and the mean connectivity (right) for various soft-thresholding power value; **(C)** Dendrogram of all genes clustered based on a dissimilarity measure (1-TOM) together with assigned module colors. **(D)** Heat map of the correlation between module eigengenes and clinical traits of TNBC. Each cell contains the Pearson correlation coefficient and p value. **(E)** Scatter plot of gene significance and module membership for blue module.

The cluster tree showed that two samples (TCGA-A1-A0SK-01, TCGA-D8-A1JF-01) deviated from the population and were eliminated in subsequent analysis steps (**Figure 7A**). The scale independence approached 0.95 when the network soft threshold was set to 8, and the mean connectivity was nearing zero. (**Figure 7B**). The transformation of the correlation matrix into an adjacency matrix was achieved *via* calculating the weighted value of the gene correlation coefficient, and the topological overlap matrix (TOM) was built. Hierarchical clustering was performed according to the constructed TOM, and different gene modules were obtained according to minModuleSize=30, labeled with different colors, resulting in a total of 28 modules,and grey modules represent genes that were not classified under any module (**Figure 7C**). We further extracted each eigengene and calculated the correlation between co-expression modules and clinical characteristics (alive, dead, and overall survival). The module’s association with overall survival (OS) was our primary focus. Results from the Clinical feature correlation heat map revealed that the black and blue modules had the highest correlation with OS (**Figure 7D**). Our research focused on the ICAM1 gene located in the blue module, a scatter plot of gene significance and module membership showed that OS is connected with genes in the blue module (r=0.2, p=3.1e-09, **Figure 7E**). We then examined the cellular pathways impacted by the genes co-expressed in the blue module. GO enrichment analysis results demonstrated that the blue module is associated with processes, such as leukocyte-mediated immunity, positive control of cytokine production, leukocyte adhesion, leukocyte activation, and monocyte differentiation (**Figure S2A**) in addition to immune receptor activity, cytokine receptor binding, cytokine activity, cytokine receptor activity, and cytokine binding (**Figure S2B**). The cellular components of the blue module include the plasma membrane, secretory granules membrane, endocytic vesicles, endocytic vesicles membrane, and phagocytic vesicles (**Figure S2C**). KEGG enrichment analysis revealed that the blue module participated in allogeneic rejection, blood cell lines, graft-versus-host disease, antigen processing and expression, and cytokine–cytokine receptor interactions. (**Figure S2D**).

### Construction of a TNBC risk score model

**Figure 8:**
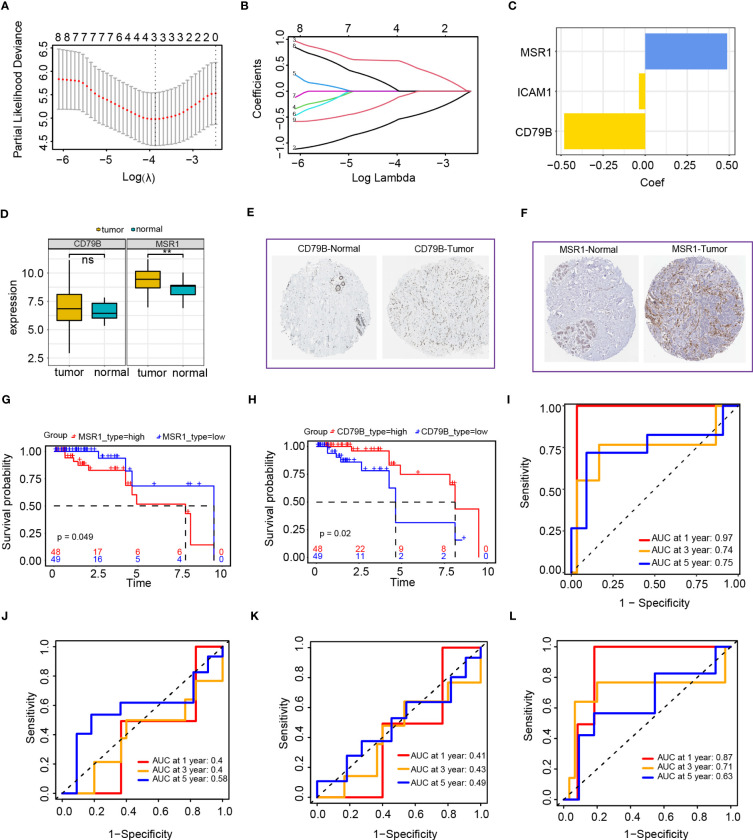
Establishment of the risk score model based on the intersection of genes in the blue module and PPI network. **(A, B)** LASSO Cox regression analysis of 2 genes; I: Cox regression coefficient of three genes; **(D)** CD79B and MSR1 genes in TNBC expression analysis **(E)** HPA database analysis of CD79B expression; **(F)** HPA database analysis of MSR1 expression; **(G)** Survival analysis of MSR1 in TNBC; **(H)** Survival analysis of CD79B in TNBC; **(I)** Time-dependent ROC curves of the risk score model; **(J)** Time-dependent ROC curves of the ICAM1 gene; **(K)** Time-dependent ROC curves of CD79B gene; **(L)** Time-dependent ROC curves of MSR1 gene.

We obtained 97 TNBC samples after excluding samples with inadequate survival information. We selected the optimal value using utilized the minimal absolute shrinkage along with selection operator (Lasso) Cox regression analysis to derive a three-gene signature (**Figures 8A, B**). The function for calculating the risk score was formulated as follows: risk score = (-0.064*ICAM1) +(-0.502*CD79B) +(0.524*MSR1), this indicates that CD79B and ICAM1 are protective factors, whereas MSR1 is a risk factor (**Figure 8C**). We analyzed the expression of levels of MSR1 and CD79B genes in TNBC and found that CD79B expression in the tumor tissues was not statistically different from the normal tissues; however, the expression levels of MSR1 were significantly elevated compared to the levels observed in the corresponding healthy tissues (**Figure 8D**). We also performed immunohistochemical validation of the CD79B and MSR1 genes employing the HPA dataset. The correlation among the expression of MSR1 and CD79B genes, as well as patient survival, was examined, and the results indicated that individuals exhibiting elevated MSR1 expression levels experienced a more unfavorable prognosis (**Figure 8E**), while those with high CD79B had a better prognosis (**Figure 8F**). We observed no difference in the CD79B expression for the tumor and normal tissues (**Figure 8G**), while MSR1 in the tumor tissues was expressed at higher levels than that in the normal tissues (**Figure 8H**). ROC curves were utilized for evaluation of the prognostic ability of the model and found an AUC of more than 0.7 at 1, 3, and 5 years (**Figure 8I**). Compared to the prognostic value of ICAM1, CD79B, and MSR1, the risk score showed superior prognostic capability than individual genes (**Figures 8J–L**).

**Figure 9:**
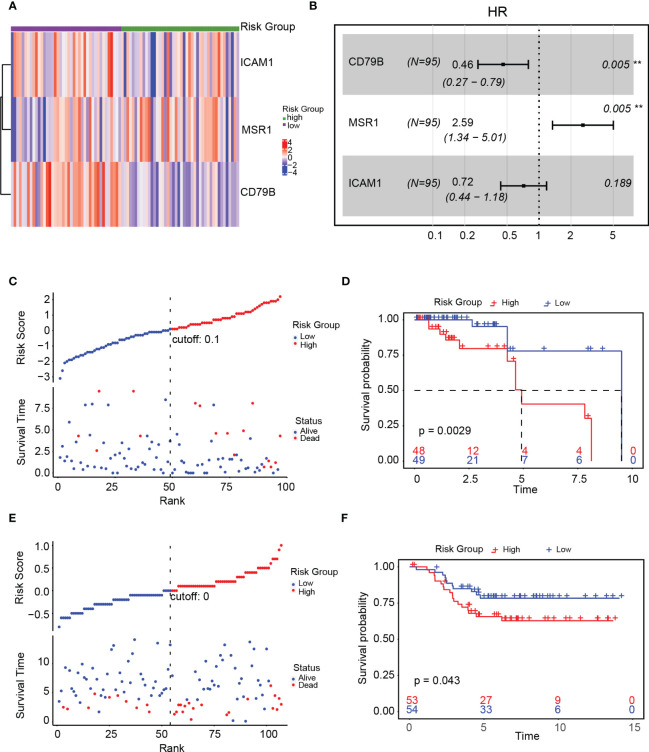
Validation of the risk score model. **(A)** Expression of ICAM1, MSR1, and CD79B in the high- and low-risk groups; **(B)** Forest plot of the gene associated with the risk score model; **(C)** Linkage diagram of the risk score model in the training set; **(D)** Survival analysis of high- and low-risk groups in the training set; **(E)** Linkage diagram of the risk factors of the risk score model in the GSE58812 dataset; **(F)** Survival analysis of the high- and low-risk groups in the GSE58812 dataset.

We created a heat map after examining the expression of ICAM1, MSR1, and CD79B genes in high- as well as low-risk groups. The high-risk group exhibited a notable upregulation of MSR1, whereas those in the low-risk group demonstrated a significant upregulation of ICAM1 and CD79B (**Figure 9A**). A forest plot was constructed to show the findings of a multivariate Cox survival analysis of the risk score model. Results indicated that ICAM1 and CD79B are protective factors (HR=0.46, p=0.003), while MSR1 is a risk factor (HR=2.18, p=0.01, **Figure 9B**). The training sets indicated that patients belonging to the high-risk group exhibited lower survival rates (**Figure 9C**). A notable difference in the duration of OS was observed among high-as well as low-risk groups (**Figure 9D**). We used the external dataset GSE58812 for the evaluation of the generalization capacity of the risk score model. The model predicted a higher death rate and shorter OS time for the high-risk group in the external data, demonstrating that the risk score model also has a strong predictive capacity (**Figures 9E, F**).

### Independent predictive value of risk score model

**Figure 10:**
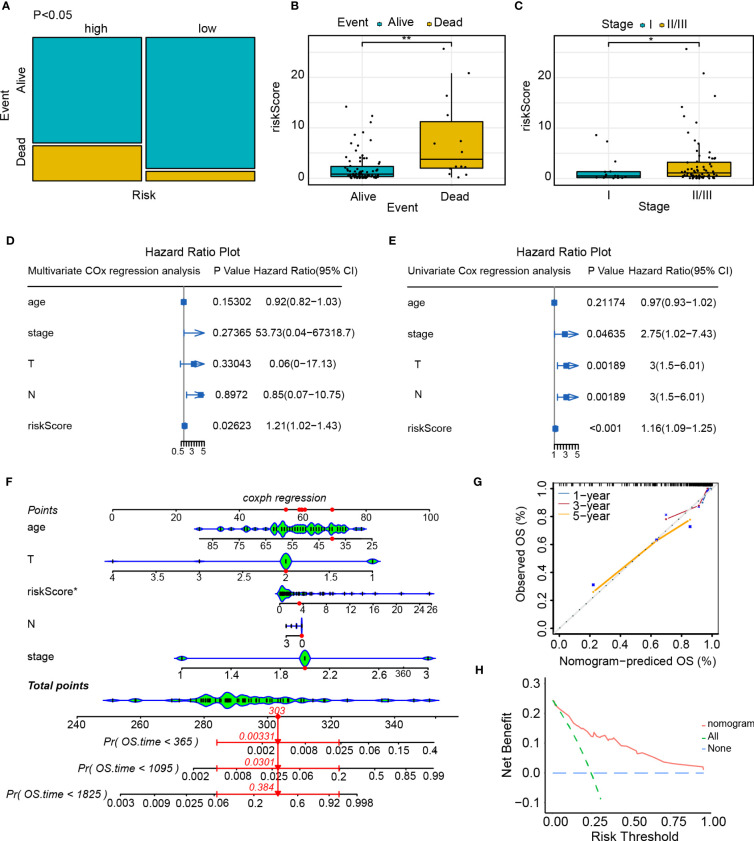
Independent predictive value of the risk score model. **(A)** Mosaic plot of the risk score and patient survival status indicators; **(B)** The correlation between the risk score and survival status of patients with TNBC; **(C)** Correlation between the risk score and clinical stage of patients with TNBC; **(D)** Univariate Cox regression analysis of the risk score and other clinical indicators; **(E)** Multivariate Cox regression analysis of the risk score and other clinical indicators; **(F)** Nomogram predicts the probability of the 1-, 3-, and 5-year OS; **(G)** Calibration curve of nomogram; **(H)** Decision curve of nomogram. *p<0.05, **p<0.01.

The contingency table chi-square test and mosaic plots were initially utilized to investigate the risk level (high and low-risk) association with survival status (dead and alive). There was a strong association between the risk level and survival status (p<0.05, **Figure 10A**). We compared the disparities in the survival status, clinical stage, and risk class and found that patients who died had a higher risk score than those who survived (live event, **Figure 10B**). Additionally, patients in stage II/III exhibited a statistically significant increase in risk score compared to those in stage I (**Figure 10C**). Univariate and multivariate Cox regression analyses were conducted to examine the potential of the risk score model as an independent prognostic factor. The results of the univariate Cox regression analysis indicate that the risk score is a prognostic factor for survival in patients with TNBC (HR=1.21, 95% confidence interval [CI]=1.02-1.43; **Figure 10D**). Upon inclusion of additional clinical parameters, such as age and stage, in the multivariate Cox regression analysis, the risk score persisted as a prognostic predictor (HR=1.16, 95% CI=1.09-1.25; **Figure 10E**). Nomogram were created for patients with TNBC, which included risk score along with clinical parameters, for survival probability prediction at 1, 3, as well as 5 years (**Figure 10F**). The calibrated curve combining the decision curve (C-index=0.72) demonstrated that the risk score model exhibited a high level of predictive capability (**Figure 10G, H**).

### High- and low-risk groups enrichment and somatic variant analysis

**Figure 11:**
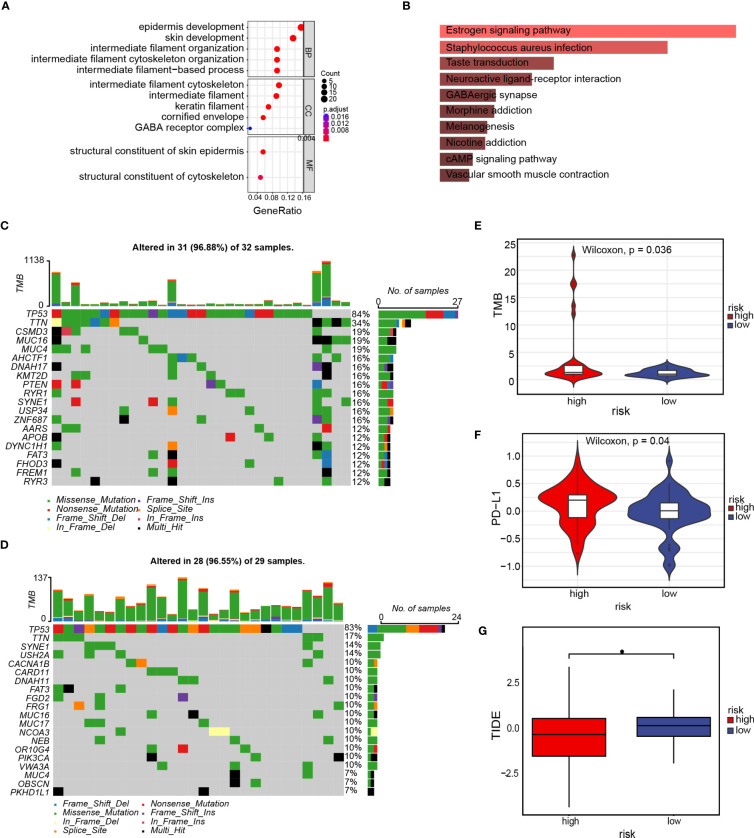
Enrichment and somatic variant analysis of the high- and low-risk groups. **(A)** GO enrichment analysis of the differentially expressed genes in the high- and low-risk groups; **(B)** KEGG enrichment analysis of differentially expressed genes in the high- and low-risk groups; **(C)** Waterfall plot displays gene mutation information with high mutation frequencies in the high-risk group; **(D)** Waterfall plot displays gene mutation information with high mutation frequencies in the low-risk group; **(E)** TMB difference in the high- and low-risk groups. **(F)** PD-L1 difference in the high- and low-risk groups; **(G)** TIDE score for the high- and low-risk groups. *p<0.05.

To evaluate differences among high- as well as low-risk groups, differential expression analysis was performed across the groups and identified 157 genes (**Table S3**). The GO enrichment analysis was done to examine the biological processes along with molecular functions of differentially expressed genes across high- as well as low-risk groups. The enriched biological processes included epidermis development, skin development, intermediate-filament organization, and intermediate-filament cytoskeleton organization. These genes were located mainly in the intermediate-filament cytoskeleton and intermediate-filament, as well as exhibited molecular functions, such as the structural component of the skin epidermis and the structural component of the cytoskeleton (**Figure 11A**). The results of the KEGG enrichment analysis (**Figure 11B**) demonstrated that the differentially expressed genes were mainly correlated with the neuroactive ligand–receptor interaction, taste transduction, *Staphylococcus aureus* infection, estrogen signaling pathway, and other metabolic pathways. We also investigated somatic mutations in the cohorts categorized as high- and low-risk, revealing a high frequency of mutations in TP53, TTN (titin), and SYNE1 (Spectrin Repeat Containing Nuclear Envelope Protein 1) in both groups. The observed mutation rate of the TTN gene was significantly greater in the high-risk group (**Figures 11C, D**). Programmed cell death ligand 1 (PD-L1), as well as tumor mutational burden (TMB), are commonly used as indicators for the evaluation of the efficacy of immunotherapy and identify populations that are likely to benefit from immunotherapy (19). As a result, we discovered that the high-risk group exhibited elevated levels of TMB and PD-L1 expression compared to the low-risk group (p=0.036 and p=0.04, respectively, **Figures 11E, F**). The Tumor Immune Dysfunction and Exclusion (TIDE) score is an index utilized for forecasting the efficacy of tumor immunotherapy. An elevated TIDE score usually indicates that patients respond poorly to immunotherapy, while a decreased TIDE score is indicative of a more favorable response. After calculating the TIDE score, we found that the TIDE scores of the low-risk group were significantly greater than those of the high-risk group, suggesting that the high-risk group exhibited a more favorable response to immunotherapy (**Figure 11G**). Based on these findings, our hypothesis proposes that immunotherapy could benefit patients in the high-risk group of TNBC.

### Immune comparison among patients in the high- and low-risk group

**Figure 12:**
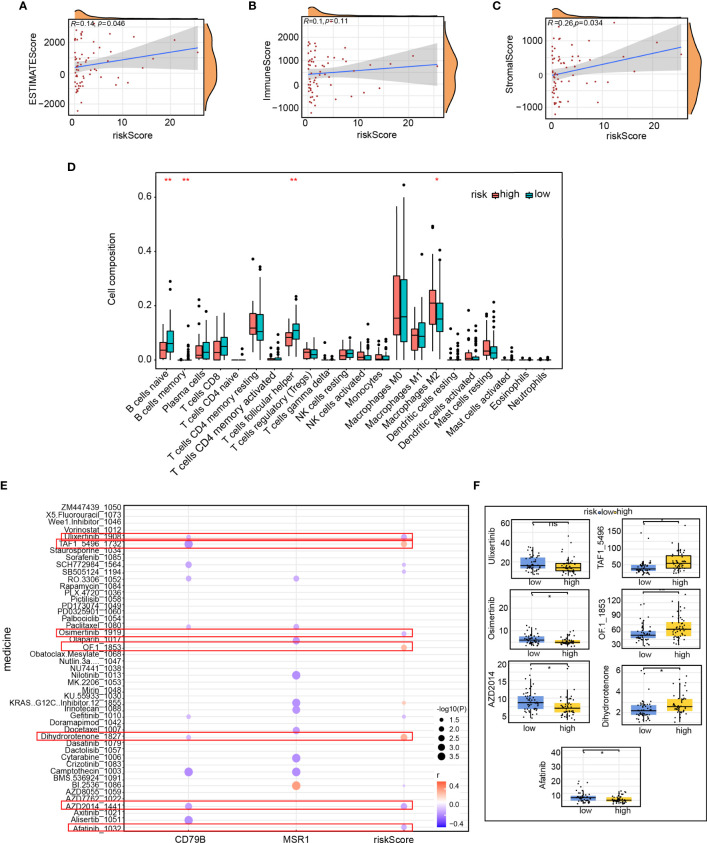
Immune comparison of patients in the high- and low-risk groups. Correlation of **(A)** ESTIMATE score, **(B)** immune scores, and **(C)** stromal scores between the risk score; **(D)** Proportion of immune infiltrating cells in the high- and low-risk groups; **(E)** Correlation between immune score and drug sensitivity predicted by the oncoPredicts algorithm; **(F)** Difference in the sensitivity of seven drugs between the high- and low-risk groups. *p<0.05, **p<0.01.

the risk score association with immune infiltration was furtherly investigated in TNBC. The purity of tumor cells is known to be correlated with the prognosis as well as tumor immune infiltration. Therefore, we employed the ESTIMATE method to analyze the correlation and calculate the immune score, stromal cell concentration, and tumor purity in patients with TNBC. We found a correlation across risk score and quantity along with the purity of tumor cells (estimated scores, R=0.14, p=0.046) and stromal cells (stromal scores, R=0.26, p=0.034, **Figures 12A–C**). However, no discernible correlation was found among the risk score as well as the immune score (**Figure 12B**). Subsequently, we examined the connection between 23 immune cells and immune scores using single-sample gene set enrichment analysis (ssGSEA). Our findings showed that the high-risk group was mostly enriched with macrophage M1, whereas the low-risk group was enriched predominantly with B-cell naïve, B-cell memory, and T-cell follicular helper cells (**Figure 12D**). To forecast the association between risk score and drug sensitivity, we used the oncoPredict algorithm and created a bubble plot to facilitate the clinical translation of the model. The graph illustrates that although the pharmaceuticals ulixertinib, osimertinib, AZD2014, and The results indicate a negative association between Afatinib and the risk score, TAF1 5496, and OF 1. Conversely, Dihydrorotenone exhibited a positive association with the risk score. (**Figure 12E**). The sensitivity differences for the seven drugs between the high-and low-risk groups are depicted in **Figure 12F**. Our findings may serve as a starting point for future clinical translations.

### Treatment decisions based on the risk score model

**Figure 13:**
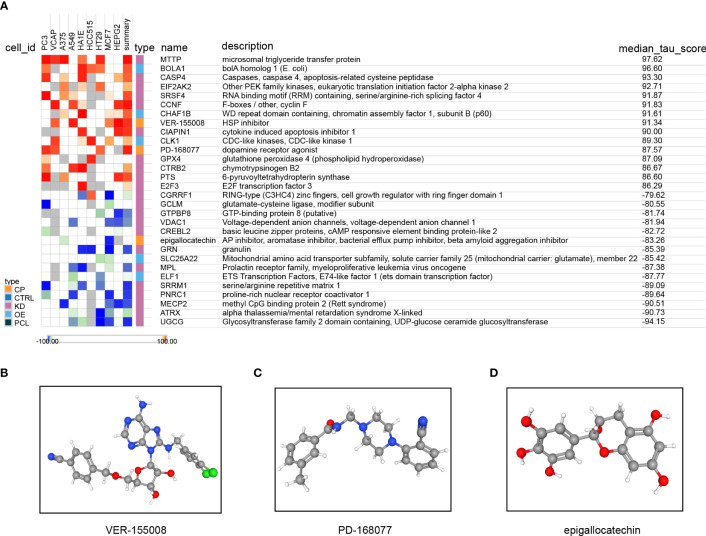
Treatment decisions based on the risk score model. **(A)** Results of the query connectivity map; the 3D structure tomographs of the three candidate small-molecule drugs for LUAD. **(B)** VEP-155008, **(C)** PD-168077, and **(D)** epigallocatechin.

A total of 157 genes were found to be differentially expressed between the high- and low-risk groups. Among these genes, 42 were observed to be down-regulated, while 115 were up-regulated. Subsequently, we used the CMap database to predict the therapeutic effects of small-molecule drugs depending on the outcomes of the differential expression analysis. According to the median tau values, ten different perturbagens, including genes and knockouts, were selected. The results suggest that, in addition to using drugs such as VEP-155008 and PD-168077, it may be beneficial to knock down or down-regulate the expression of MTTP, CASP4, EIF2AK2, SRSF4, CCNF, CIAPIN1, GPX4, CTRB2, PTS, and E2F3 for improvement of TNBC patient’s prognosis. The prognosis was found to be poorer when genes such as CGRRF1, GCLM, GTPBP3, VDAC1, CREBL2, GRN, MPL, SRRM1, PNRC1, MECP2, ATRX, or UGCG were knocked down or downregulated. Additionally, patients had a poorer prognosis due to the use of epigallocatechin drugs, overexpression of the Solute Carrier Family 25 Member 22 (SLC25A22) and E74 Like ETS Transcription Factor 1 (ELF1) genes, and other factors (**Figure 13A**). The 3D structures of the small molecule drugs VEP-155008, PD-168077, and epigallocatechin were analyzed using the PubChem database (**Figures 13B–D**).

### Low expression of ICAM1 promotes polarization of M2 macrophages and T-cell exhaustion

**Figure 14:**
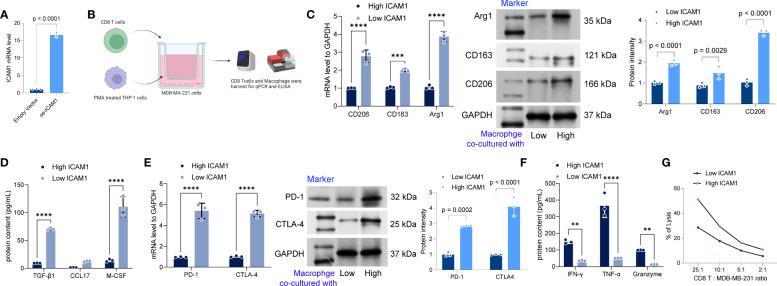
Low expression of ICAM1 promotes polarization of M2 macrophages and T-cell depletion. **(A)** The overexpression of ICAM1 in MDB-MA-231 cells *via* Lipofectamine2000 mediated transfection. **(B)** schematic diagram of the co-culture of low- and high ICAM1 MDB-MA-231 cells with PMA-treated THP-1 cells and CD8 T cells; **(C)** Expression levels of CD206, CD163, and Arg1 increased significantly in the cells after co-culture based on the qRT-PCR assay and Western blot; **(D)** ELISA to detect the levels of TGF-β1, CCL17, and M-CSF in the cell culture medium; **(E)** qRT-PCR and western blot to detect the mRNA expression levels of PD-1 and CTLA4 in CD8 T cells after co-culture with low- and high ICAM1 MDA-MB-231 cells; **(F)** ELISA to detect the levels of IFN-gamma, TNF-α, and granzyme levels. **(G)** The LDH assay kit is used to detect the cytotoxic effect of CD8 T cells on MDA-MB-231 cells. Data are presented as mean ± SD and statistically analyzed using 2-way ANOVA, **p<0.01, *** <0.001, **** <0.0001.

In our previous study, we revealed that individuals exhibiting reduced ICAM1 expression experienced a decrease in their OS duration and worse anti-PD-L1 drug sensitivity. Furthermore, we performed ICAM1 overexpression in MDB-MA-231 cells, where cells transfected with the Empty vector represented the Low ICAM1 group, while those transfected with the ICAM1 overexpression plasmid represented the High ICAM1 group (**Figure 14A**). Thus, we speculated whether the low expression of ICAM1 altered the tumor microenvironment in TNBC patients. To validate this speculation, we used TNBC cells with low ang high ICAM1 MDB-MA-231 cells and co-cultured them with PMA-treated THP-1 cells and CD8 T cells, respectively. We found that after co-culturing with macrophages, the polarization ratio of M2-type macrophages increased significantly, and the levels of tumor-promoting cytokines TGF-β1, CCL17, and M-CSF were significantly increased (**Figures 14B–D**). However, TNBC cells with low ICAM1 expression co-culturing with CD8 T cells to study the alteration of ICAM1 expression in the tumor microenvironment of TNBC patients, the expression levels of PD-1 as well as CTLA4 were substantially elevated, and the levels of IFN gamma, TNF-α, and granzyme were significantly decreased (**Figures 14E, F**). Moreover, we have included the results of the LDH assay to evaluate the cytotoxic effects of CD8 T cells on MDB-MA-231 cells. The results demonstrated that low ICAM1 MDD-MA-231 cells were less sensitive to CD8+ T cells (**Figure 14G**). So, low expression of ICAM1 may be associated with immune escape in TNBC.

## Discussion

TNBC is a subtype of breast cancer that exhibits high aggressiveness, leading to a poor prognosis and a tendency for early recurrence (20, 21). The overall survivals of most patients with metastatic TNBC after systemic chemotherapy is less than 18 months (22). Therefore, there is a need for new treatments to prolong survival. In recent years, patient survival in other solid tumors has been prolonged with immunotherapy. Immune checkpoint inhibitors are commonly used immunotherapy medications that boost the cytotoxicity and proliferation ability of tumor-infiltrating lymphocytes by blocking the binding of inhibitory receptors (including CTLA-4 and PD-1) (23). TNBC may benefit from immunotherapy more than other breast cancer subtypes due to a higher PD-L1 expression in tumor and immune cells as well as a higher TMB. With an increase in TMB, the number of tumor-specific neoantigens displayed on the surface of tumor cells also increases. These neoantigens activate neoantigen-specific T lymphocytes, triggering anticancer immune responses (24–26). New biomarkers in immunotherapy responses need to be identified to improve the therapeutic impact and thus quality of life. The immune system and inflammatory responses of the body are influenced by ICAM1. It may also facilitate tumor cell attachment to different cell types, enabling malignancies to evade immune monitoring. Tumor incidence and growth are correlated to ICAM1 expression (27). ICAM1 gene was the focus of our study. Pan-cancer expression analysis revealed that ICAM1 is expressed differentially in cancer types, and ICAM1 expression was up-regulated in TNBC.

ICAM1 was reported to be related to the prognosis of different tumors (28). The present study aimed to examine the correlation between the expression of ICAM1 and TNBC prognosis and found that ICAM1 expression was associated with Disease Free Survival (DFS). However, this variation did not achieve statistical significance. The DFS rate of people having low ICAM1 expression was poorer than those with high ICAM1 expression. The inadequate sample size is the cause of the low 5-year survival rate in breast cancer. Research has indicated that a reduction in ICAM1 expression results in diminished cancer cell adhesion and promotes distant metastasis of cancer cells (29). It is speculated that low ICAM1 expression enhances the metastatic potential of TNBC and reduces the survival time of patients. Our investigation of the relationship between ICAM1 expression and clinical characteristics revealed that ICAM1 levels were low at higher clinical stages of TNBC. This outcome is consistent with the prognosis correlation. The expression level of ICAM1 decreases as TNBC progresses, and this has a negative impact on the prognosis. Mutation analysis found that the non-synonymous mutation T231M in ICAM1 reduced the hydrogen bonds between the main chains of K246 and weakened the interaction. It is speculated that it may affect the stability of the ICAM1 protein. A search of the COSMIC database showed that the T231M mutation is only found in breast cancer and may impact immune system signaling, however, no other studies have reported this mutation. Moreover, Individuals belonging to the high-risk group exhibit significantly elevated mutation rates in TP53, TTN, and SYNE1 genes, along with increased TMB and PD-L1 levels and decreased TIDE scores. Studies have shown that p53, a tumor suppressor protein, can regulate the expression of ICAM1. Activation of p53 in response to cellular stress or DNA damage can lead to increased ICAM1 expression. This upregulation of ICAM1 may promote immune cell recruitment and facilitate the anti-tumor immune response (30). ICAM1 expression on tumor cells or surrounding stromal cells can modulate immune cell interactions and affect the infiltration of cytotoxic T cells into the tumor. Conversely, p53 mutations or dysfunction can lead to immunosuppression or evasion of immune surveillance. The interplay between ICAM1 and p53 in the tumor microenvironment can impact tumor progression, immune responses, and patient outcomes (31, 32).

The relationship between ICAM1, TTN (Titin), and SYNE1 (Spectrin Repeat Containing Nuclear Envelope Protein 1) in cancer is not extensively studied, and their direct interplay in cancer is not well established. SYNE1 has been implicated in the regulation of gene expression through its involvement in chromatin organization and nuclear envelope dynamics. Altered gene expression patterns can influence the tumor microenvironment by affecting immune cell infiltration, extracellular matrix remodeling, and angiogenesis (33). Dysregulation of SYNE1 or related proteins may therefore contribute to changes in the tumor microenvironment through indirect effects on gene expression program (34). The tumor microenvironment is characterized by altered mechanical properties, such as increased tissue stiffness. TTN, being a protein involved in muscle elasticity, may contribute to the mechanical properties of cancer cells or the surrounding stroma. Changes in TTN expression or function within the tumor microenvironment could potentially influence the mechanical interactions between cancer cells, the extracellular matrix, and surrounding stromal cells (35). Besides, TTN can interact with various signaling molecules and influence signal transduction pathways. In the tumor microenvironment, signaling pathways play a crucial role in cell survival, proliferation, and interactions with the surrounding stroma. Changes in TTN expression or alterations in its interaction with signaling molecules could potentially impact cellular signaling events within the tumor microenvironment (36).

The protein interaction network has shown that ICAM1 participates in leukocyte cell adhesion, leukocyte mobility, and leukocyte-related immune activation. Analyzing the variations within the high-ICAM1 as well as low-ICAM1 groups suggests that ICAM1 is involved in regulatory processes, such as leukocyte cell adhesion, mononuclear cell differentiation, control of T-cell activation, and positive regulation of leukocyte cell adhesion. Moreover, high-ICAM1 groups outperformed stromal cells (p=0.037), tumor cell purity (p=3.8e-10), and immune score (p=5.4e-08). The expression of ICAM1 correlated with common immune checkpoint inhibitors in the ICAM1 low-expression group. ICAM1 may thus be used as a molecular marker in TNBC treatment.

M1 macrophages are immunological cells with tendencies in existence in a polarized state. When macrophages are stimulated, they develop into M1 macrophages and exhibit pro-inflammatory and cytotoxic activities. In the early stage of lung cancer, M1 macrophages can recognize and remove tumor cells and play a role in immune surveillance (37). In addition, M1 macrophages can induce and enhance the immune response of T cells, thus enhancing the cytotoxic effect on tumors (38). However, the role of M1 macrophages may shift to promoting tumor growth and metastasis when lung cancer progresses to advanced stages (39). The study conducted a comparison of immune cell infiltration between high- and low-risk groups in TNBC. The findings revealed that the high-risk group exhibited a greater abundance of M1 macrophages. Thus, combining findings from previous studies with our analysis, we suggested that M1 macrophages in the high-risk set promoted TNBC cell growth and metastasis.

The WGCNA approach is based on a network with a scale-free structure, which examines the gene expression patterns of many sample genes. The genes are clustered according to the expression patterns, and the clustered gene set is called a module. The correlation between the module and clinical information can be analyzed, and the key module can be identified (40). The WGCNA analysis was used in this study to identify a total of 28 modules based on the TNBC gene expression data. The association between each module and the patient’s OS time was also examined. Among them, the black and blue modules significantly correlated to patient survival time, and ICAM1 belonged to the blue module. The genes in the blue module are involved in leukocyte-mediated immunity, cytokine production positive regulation, leukocyte cell adhesion, as well as other biological processes, suggesting that these co-expressed genes are implicated in the immunology of TNBC. The risk score model is often employed to examine survival outcomes related to patients’ disease characteristics (41).

According to the genes intersection of the blue module and the PPI network, a risk score model comprising ICAM1, MSR1, and CD79B was constructed using Lasso Cox regression analysis. Several studies have shown that the MSR1 gene may be involved in tumorigenesis and development and that the level of MSR1 expression in tumors is closely related to malignancy and prognosis. For example, reduced expression levels of MSR1 in tumors, such as lung cancer and melanoma, may be associated with invasion, metastasis, and poor prognosis. While in other tumors, such as colorectal cancer, a high expression of MSR1 may be associated with poor prognosis. We also found that high MSR1 expression in TNBC had a poorer prognosis (42–44). The expression levels of the CD79B gene were associated with prognosis in various cancers. In gastric and non-small cell lung cancer, a decreased CD79B expression was related to shorter survival as well as tumor malignancy (45, 46). Thus, CD79B is a protective factor in gastric and non-small cell lung cancers, which corroborated the finding that TNBC patients exhibiting elevated levels of CD79B expression demonstrated a more favorable prognosis. The patients were split into two distinct groups, which are high- and low-risk according to risk score function. The TCGA and GEO datasets were employed to compare the survival outcomes of the two groups. The results indicated that the high-risk group had a shorter OS time and higher mortality rate. The independent prognostic capability of the risk score model was demonstrated by both univariate and multivariate Cox regression analyses. The risk score model showed a strong predictive performance based on the time-dependent ROC analysis, a nomogram was created to forecast the likelihood of patients’ survival in the future. Significantly, the differential expressions of genes in the high- and low-risk groups with TNBC had an influence on biological processes, such as the development of the epidermis and skin and the construction of intermediate filaments in the cytoskeleton. The differentially expressed genes are believed to be linked to the epidermal growth factor (EGFR) signaling pathway. Moreover, the high-risk group had substantially increased TMB and PD-L1 expression levels and lower TIDE scores than the low-risk group.

As we investigated the relationship between the risk score and medication sensitivity further, we discovered that the risk score had a favorable association with TAF1 5496, OF 1, and dihydrorotenone and an unfavorable correlation with ulixertinib, osimertinib, AZD2014, and afatinib. These findings lead to the hypothesis that immunotherapy may benefit high-risk group patients. To better decide treatment strategies for patients with TNBC, a link between drug, gene, and disease was found through changes in gene expression, and the analysis was completed using the CMap database. The results showed that the knockdown of genes, such as MTTP, CASP4, EIF2AK2, and SRSF4, and the use of VEP-155008 and PD-168077 improved the prognosis in patients with TNBC. In recent years, VER-155008 has gained increased research focus in tumor therapy. Several studies have shown that VER-155008 has anti-tumor effects on cancers, such as colorectal, lung, and breast (47, 48). Our results showed that VER-155008 improved the prognosis of patients with TNBC. PD-168077 has gained increased attention in the treatment of obesity and metabolic diseases. Several studies have shown that PD-168077 can improve insulin resistance and secretion and lower blood glucose levels by activating GPR40 (49). There are no reports on the use of PD-168077 in oncology treatment. Our finding that PD-168077 can be used to improve TNBC patients’ prognosis requires additional preclinical as well as clinical studies to understand its efficacy and safety. However, the knockdown of CGRRF1, GCLM, GTPBP3, and VDAC1 and the use of epigallocatechin led to a worse prognosis for patients. Epigallocatechin has been investigated as a possible adjuvant therapeutic agent. Research findings have indicated that it can prevent the proliferation and spread of cancer cells through pathways, such as regulation of apoptosis, cell cycle, and tumor angiogenesis (50). However, our study suggests that epigallocatechin may not benefit patients with TNBC. When TNBC cells with low expression of ICAM-1 were co-cultured with PMA-treated THP-1 cells and CD8 T cells, respectively, a substantial rise in polarized M2-type macrophage proportion was observed. an immunosuppressive macrophage subtype that can inhibit immune cell activation and promote tumor growth (51). This may be related to the fact that TNBC cells low in ICAM-1 expression inhibits the immune cells’ function in the tumor microenvironment and induce polarization of M2-type macrophages. In addition, the expression of PD-1 and CTLA4 may increase when low ICAM-1 expressing TNBC cells are co-cultured with CD8 T cells, while secreted immune effector molecules are reduced, suggesting that CD8 T-cells may be suppressed. Thus, low expression of ICAM1 promotes the polarization of M2 macrophages as well as T-cell exhaustion.

Interestingly, we found that ICAM1 levels gradually decrease with the progression of TNBC. This can cause an increase in tumor cell invasion and metastasis, allowing evasion of immune surveillance and thus negatively impacting patient prognosis. In contrast, in prostate cancer, tumor cells evade NK cell attack by suppressing ICAM1 expression (52). Theranostic nanoparticles modified with anti-ICAM1 target could specifically target TNBC in an *in vitro* experiment by Chen et al. (53). However, a recent study found that ICAM1-deficient breast cancer cells develop large metastatic lesions (54). Thus, the use of anti-ICAM1 targets to treat tumors may be accompanied by an increased risk of potential metastasis.Therefore, the clinical significance of anti-ICAM1 approaches may be further enhanced through personalized medicine approaches. Identifying patient subgroups with elevated ICAM1 expression or specific genetic profiles associated with ICAM1 regulation can help tailor treatment strategies and improve therapeutic responses. However, ICAM1 has been implicated in the metastatic spread of cancer cells. By promoting interactions between tumor cells and endothelial cells, ICAM1 facilitates the adhesion and transmigration of cancer cells to distant organs. Hence, there’s still the complexities and potential limitations of anti-ICAM1 approaches.

There are still some limitations of our study. Firstly, the TCGA database for BRCA included only a small number of TNBC cases. The analysis comprised a sample of fewer than 100 patients, following the exclusion of samples that had incomplete survival data. Secondly, when we analyzed the sub-cellular localization of ICAM1, only breast cancer samples could be selected because no TNBC samples related to ICAM1 expression were retrieved from the HPA database; this may affect the analysis results. Nevertheless, the limited sample size lacks conclusive evidence on whether the up-regulation of ICAM1 is closely related to the increase in TNBC metastases, high recurrence rate, and OS, additional clinical studies are warranted in this direction.

In conclusion, the ICAM1 expression level is correlated with the TNBC prognosis. Low ICAM1 expression may be related to immune escape, leading to poor treatment response and worsened prognosis. ICAM1 has the potential to function as a prognostic indicator as well as a treatment target for TNBC.

## Data availability statement

The original contributions presented in the study are included in the article/**Supplementary Material**. Further inquiries can be directed to the corresponding author.

## Author contributions

QZ: Conceptualization, formal analysis, funding acquisition, and writing the original draft. JX: Investigation, data curation, and visualization. YX: Investigation, formal analysis, and data curation. SS: Methodology and software analysis. JC: Writing review and editing. All authors contributed to the article and approved the submitted version.
