# Supplementary material for: Role of ICAM1 in tumor immunity and prognosis of triple-negative breast cancer
Source: Front Immunol. 2023 Aug 21;14:1176647. doi: 10.3389/fimmu.2023.1176647 (PMC10475526; doi:10.3389/fimmu.2023.1176647)
Supplement: Supplementary file 1 [file DataSheet_1.pdf]

Supplementary Material

Role of ICAM1 in tumor immunity and prognosis of triple-negative breast cancer

Qin Zhou[1,2], Jiawei Xu[2], Yan Xu[2], Shaokun Sun[2], Jian Chen[1,2*]

[1]Suzhou Medical College of Soochow University, Suzhou, 215008, China;

[2]Department of Breast surgery, Affiliated Kunshan Hospital of Jiangsu University, Kunshan, 215399, China.

[*]**Corresponding Author**: Jian Chen, Eamil:chen_jian818@163.com.

1 Supplementary Figures and Tables

1.1 Supplementary Figures

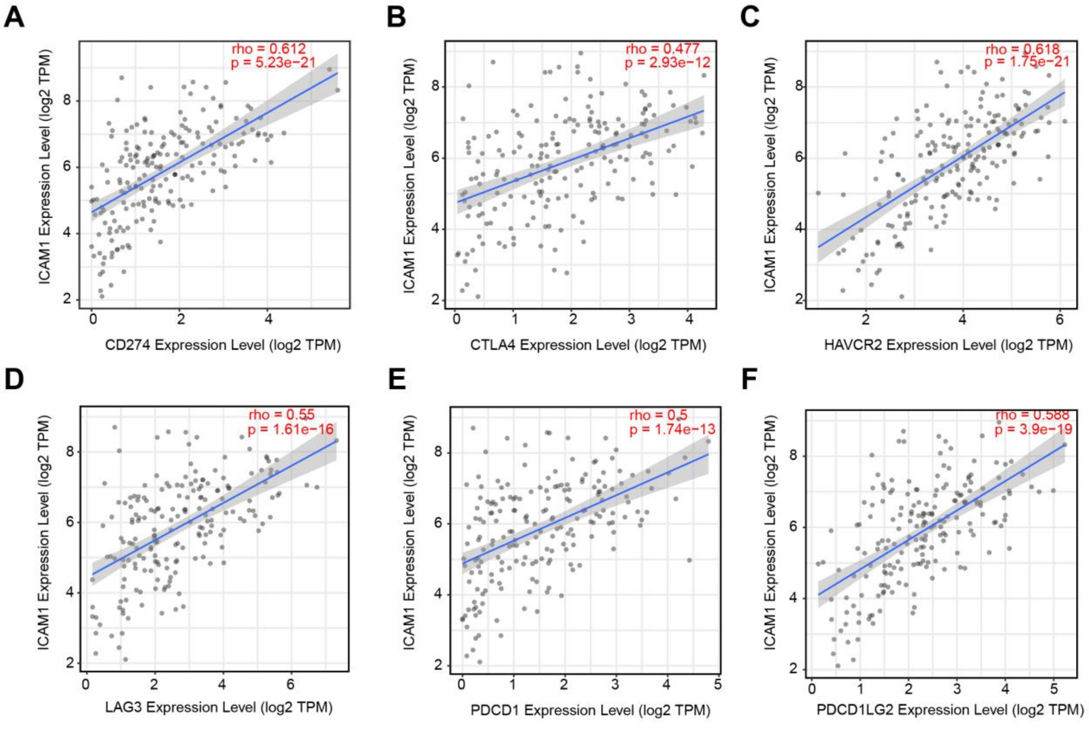

Fig S1. Correlation between ICAM1 and immune checkpoint molecules. They are (A): CD274; (B): CTLA4; (C): HAVCR2; (D): LAG3; (E): PDCD1; (F): PDCD1LG2.

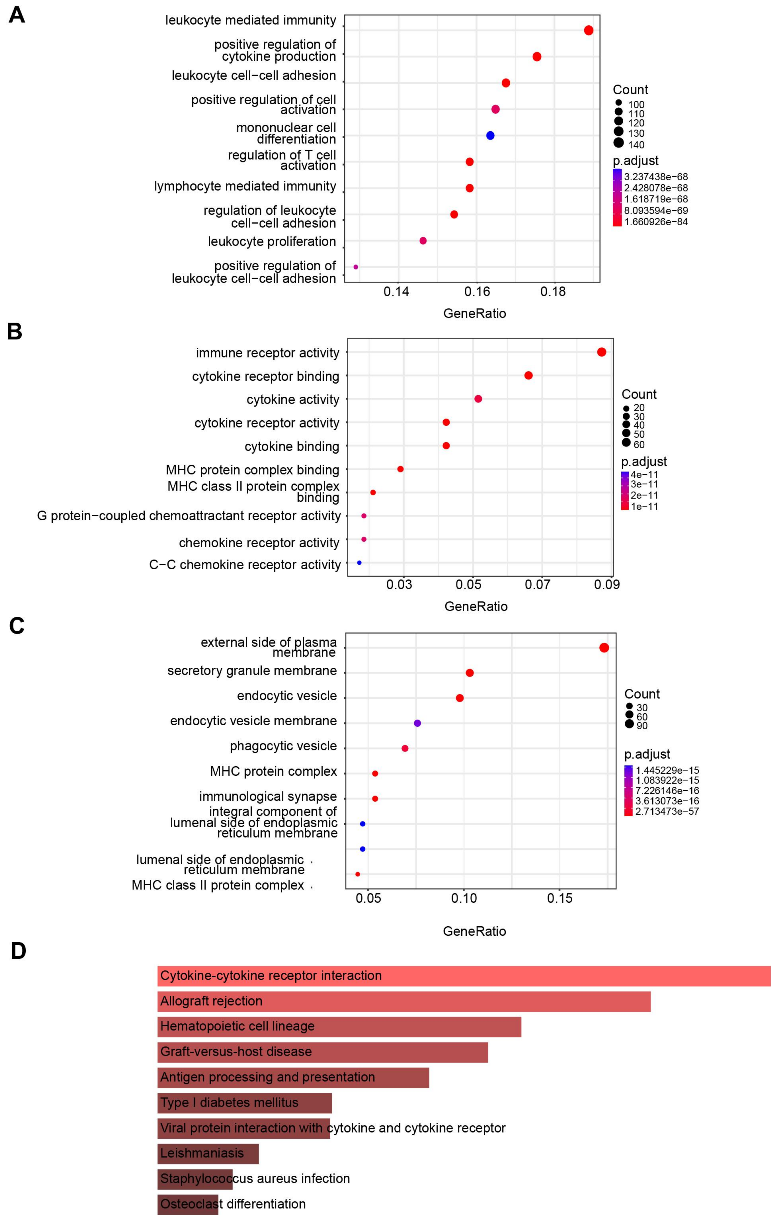

Fig S2. Gene enrichment analysis in the blue module. (A): Biological processes (BP) enrichment analysis; (B): Molecular function (MF) enrichment analysis; (C): Cellular components (CC) enrichment analysis; (D): KEGG enrichment analysis.

1.2 Supplementary Tables

Table S1. The abbreviations and the full name of analyzed tumors in the current study

Abbreviation	Tumor name
LUAD	lung adenocarcinoma
SKCM	skin cutaneous melanoma
LUSC	lung squamous cell carcinoma
KICH	kidney chromophobe
BRCA	breast invasive carcinoma

CHOL	cholangiocarcinoma
HNSC	head neck squamous cell carcinoma
KIRC	kidney renal clear cell carcinoma
KIRP	kidney renal papillary cell carcinoma
STAD	stomach adenocarcinoma
THCA	thyroid carcinoma
UCEC	uterine corpus endometrial carcinoma

Table S2. Results of differential analysis of TNBC patients with high and low expression of ICAM1.

Gene	logFC	AveExpr	t	P.Value
ANXA7	-1919.666667	8851.833333	-4.271207495	0.000525536
RABEP2	-888.1111111	2335.833333	-4.108212078	0.000745935
FLOT1	-4724.777778	10916.94444	-4.077662631	0.000796659
CDK14	1055.444444	1654.5	4.030599929	0.000881702
ZNF322A	-829.4444444	2262.944444	-3.989612394	0.000963196
RNF5	-1571.444444	2623.944444	-3.954960403	0.001037989
SNRNP35	-581.5555556	1148.888889	-3.900323086	0.001167974
ADAM9	2911	5229.722222	3.870409606	0.001245951
ZNHIT3	-1193.111111	2188.444444	-3.823538884	0.00137878
H3F3B	-16630.55556	37351.16667	-3.81905296	0.001392216
PGAP1	370.2222222	715.7777778	3.799484322	0.001452377
SPPL3	-871.8888889	3817.611111	-3.789081851	0.001485412
MEA1	-1150.333333	3259.944444	-3.788871325	0.001486088
DOCK10	1519	1732.277778	3.780447931	0.001513402
RBM38	1436.777778	2036.611111	3.748310248	0.001622313

STK40	1713.333333	3484.444444	3.740742368	0.001649082
C5orf30	-1360	2398.777778	-3.710722689	0.001759691
B4GALT5	2327.444444	4919.166667	3.689279913	0.001843213
AKIRIN1	850	3097.111111	3.668750742	0.001926888
AP3S2	-2040.333333	4919.166667	-3.662754041	0.001952038
FIG4	-1030.333333	1955.5	-3.646337518	0.002022581
EHD1	2929.666667	5270.277778	3.587955237	0.002294703
COX6C	-48134.77778	42273.27778	-3.587034707	0.002299274
SULT2B1	-749.1111111	698.2222222	-3.548276895	0.002500183
RANGRF	-577.5555556	1043.555556	-3.502372575	0.002760854
STK35	1474.333333	3587.944444	3.475404982	0.002926411
PDSS2	-658.6666667	1110.777778	-3.454869221	0.003059063
ZNF428	-987.8888889	1949.833333	-3.433058941	0.003206486
TCEA1	-1636.222222	4610.777778	-3.407953868	0.003384921
CREB3L2	3177.555556	5905.555556	3.358053012	0.003769309
NDUFA13	-3083.444444	6186.277778	-3.335135732	0.003960021
TOMM6	-2362	6553.444444	-3.334110938	0.003968768
SLC35A1	-1010.444444	1951.777778	-3.315869397	0.004127706
CRY1	-448.3333333	1271.944444	-3.301260911	0.004259509
TMEM199	-523	1629.166667	-3.291580234	0.004349132
PSMD9	-1104.111111	2568.055556	-3.263165817	0.004623073
PEBP1	-8118.666667	22345.33333	-3.255439052	0.004700463
PLCH2	618.1111111	483.1666667	3.250909511	0.004746423
PLD1	421.4444444	610.0555556	3.25013713	0.004754304

PPP1R2	-1375.888889	4227.833333	-3.249199411	0.004763889
ABCA2	2203	5341.722222	3.243573085	0.004821804
PACSIN3	-2231.333333	2786	-3.238989527	0.004869496
RNF19B	1073	2019.166667	3.236469396	0.004895916
ZDHHC6	-552.2222222	2298.777778	-3.227304731	0.00499319
EML3	533.1111111	2421.777778	3.204606322	0.005242375
SYVN1	2501.777778	5147	3.200982004	0.00528328
ZNF193	-482.2222222	725.4444444	-3.191899243	0.005387174
RWDD1	-1127.222222	2638.5	-3.190362781	0.005404947
ANP32A	-3842.444444	8788.444444	-3.142441722	0.005989111
SEC61A1	5994	23567.33333	3.14140973	0.006002349
ZUFSP	-209.2222222	565.8333333	-3.12735077	0.006185584
PCDHA4	-551.3333333	322.5555556	-3.104817114	0.006490769
ICAM1	4084.888889	3639.777778	3.091421664	0.006679129
CD164	-13885.44444	28051.61111	-3.083133514	0.006798341
TRAPPC5	-820.2222222	1744.777778	-3.054628382	0.007224447
KLHL3	199.7777778	298.5555556	3.047868673	0.007329261
DNAJC17	-344.4444444	666.1111111	-3.043920615	0.007391164
GTF3C6	-1454.111111	2494.166667	-3.04328187	0.007401227
ANAPC7	-1312.222222	3385.222222	-3.039062571	0.007468037
IFT20	-405.3333333	1125.333333	-3.034971134	0.007533384
IL6R	295.8888889	383.6111111	3.034727611	0.007537291
MAP4	4229.222222	16745.72222	3.030601106	0.007603797
MYLIP	-1458.555556	2744.944444	-3.029489473	0.00762181
PALM	-1482.444444	2064.666667	-3.019288709	0.007789067

TNKS1BP1	8406.333333	19069.05556	3.018343501	0.007804745
C12orf47	-323	773.5	-3.013908184	0.007878726
IL1RAP	519	672.6111111	2.998207754	0.008146144
FAM50A	-919.3333333	3585.111111	-2.988202477	0.008321152
GANAB	12512.55556	28093.05556	2.987521632	0.008333193
SPG21	-1964	6243.333333	-2.984471302	0.00838735
BLVRB	-2885.555556	4869.333333	-2.980151912	0.008464624
JAK3	811.8888889	810.9444444	2.978843468	0.008488169
SFRS8	-651.6666667	2401.722222	-2.977991588	0.008503532
C2orf64	-651.2222222	1469.611111	-2.975082066	0.008556208
GOT1	-1109.222222	2606.5	-2.968732027	0.008672281
AEN	-791.3333333	1404.444444	-2.961864982	0.008799528
SLC19A1	-584.3333333	1451.611111	-2.958147017	0.008869179
ELF4	1209.222222	1750.388889	2.957931113	0.00887324
PFKP	2108.111111	2629.5	2.957867135	0.008874443
SCYL1	1692.888889	6240.111111	2.955173183	0.008925275
EXOSC7	-491.8888889	1500.833333	-2.941606533	0.009185593
SRP14	-5273.444444	12102.83333	-2.926038609	0.009493409
PPT2	-300.7777778	773.2777778	-2.919871197	0.009618105
ANXA9	-1675	1892.833333	-2.919719383	0.009621195
NEK6	977.7777778	2374.444444	2.916654698	0.009683768
HIST1H4J	-153.3333333	165.6666667	-2.908374219	0.009854815
ZBTB46	444	741.4444444	2.901354684	0.010002109
SAMD4A	578.4444444	810.8888889	2.898713793	0.010058075
UBE2N	-1750.777778	5753.611111	-2.889144937	0.010263413

LRIG3	501	750.6111111	2.88177913	0.010424238
TNC	29077.33333	29063.55556	2.879681578	0.010470481
COX6A1	-3994.444444	6994	-2.876820795	0.010533871
ABCF3	-668	3015.111111	-2.873797977	0.010601255
INTS5	762.7777778	2287.944444	2.870462447	0.010676095
STAU1	3064.555556	10962.05556	2.86877263	0.010714204
NEU1	-2696.333333	5570.722222	-2.861730351	0.010874448
RNF114	2127.888889	5316.055556	2.855782559	0.011011592
PDE4DIP	4164.888889	7384.555556	2.847856695	0.01119695
ZBED1	1297.777778	4057.888889	2.843556029	0.011298787
KCTD9	794.3333333	1632.722222	2.839015174	0.011407284
SUDS3	-1116.444444	4530.222222	-2.838766367	0.011413258
ZNF267	500.2222222	1246.333333	2.835661827	0.011488052
RPP21	-699.4444444	1271.833333	-2.833424554	0.011542246
RFXANK	-845.6666667	2552.388889	-2.833367253	0.011543638
ZNF33A	-1140.555556	3569.277778	-2.831571173	0.011587328
CAPN1	8502.888889	17066	2.830955968	0.01160233
CARD9	407.6666667	373.9444444	2.829538856	0.011636958
SCAMP2	-1362.888889	5872.444444	-2.828938786	0.011651652
IL32	3436.555556	3515.277778	2.823233094	0.011792258
NNMT	2521	4382.055556	2.821871823	0.011826046
ADCY3	1813.222222	2728.944444	2.821680639	0.011830799
TGFB1	1731.888889	3969.833333	2.816519384	0.011959805
ARPC1B	4475.333333	8819.666667	2.815946106	0.011974218

MAD1L1	-348.2222222	1046.111111	-2.811690557	0.012081732
ARHGAP9	242.3333333	366.5	2.811393548	0.012089271
SERPING1	10996.22222	13200.44444	2.810826044	0.012103687
SAP18	-3255.111111	7985.888889	-2.809170325	0.012145843
CMIP	2714.888889	3571.777778	2.806945627	0.012202709
SLC2A1	2608.333333	5060.5	2.80134813	0.012346926
TCEAL4	-10500.88889	11435.88889	-2.800606552	0.012366155
QRSL1	-877	1411.5	-2.798322054	0.012425575
TRIAP1	-667.3333333	1593.666667	-2.79548918	0.01249964
NOC4L	-627.8888889	1451.388889	-2.792781104	0.012570841
CANT1	-2118.777778	7054.833333	-2.790711128	0.012625528
ATP6V0E1	-3291.888889	9817.722222	-2.788819078	0.012675715
PEX2	-1089.555556	3115.555556	-2.787843142	0.012701677
ITGAV	8861.888889	12012.94444	2.784968775	0.01277844
AKNA	1222.222222	1990.888889	2.779867059	0.012915788
POLR1A	721.5555556	2015.444444	2.777577169	0.012977898
VPS52	-970.3333333	4467.722222	-2.776674958	0.013002449
CTSB	22141.22222	47994.27778	2.775424331	0.013036554
COX7A2	-3124	5247	-2.772588752	0.0131142
EGFR	1081	794.1666667	2.766946837	0.013270019
MED14	2118.222222	4585.444444	2.766846462	0.013272808
APOBEC3C	422	543.2222222	2.762300292	0.013399685

VPS8	-528.2222222	2486.111111	-2.757401715	0.013537705
AMPD3	456.4444444	866.6666667	2.754810823	0.013611257
ATP6V1E1	-1449.555556	5780.777778	-2.754388867	0.013623272
DLGAP4	1688	5854.666667	2.753293062	0.013654522
LTBP3	3744.888889	8925.222222	2.752874347	0.013666482
SCARA3	2600.888889	2567	2.745074543	0.013891108
RTN4IP1	-321.1111111	489.6666667	-2.742473384	0.013966805
DPYSL2	2562.111111	4914.055556	2.740644658	0.014020261
SLC35B3	-436.7777778	1631.944444	-2.739339914	0.01405852
XPOT	988.5555556	5226.722222	2.735109722	0.014183254
DNAJB9	861.1111111	2228.444444	2.733374075	0.01423474
PACS1	2777.888889	6375.055556	2.731242846	0.014298205
MAN2B1	2525.222222	6474.166667	2.727880222	0.014398893
LRRC8E	-699.5555556	1206	-2.726893989	0.014428554
ZFP90	778.7777778	2120.055556	2.717662372	0.014709047
TLR2	972.2222222	1130.111111	2.71514047	0.014786578
SFRS9	-2746.111111	7943.611111	-2.710159122	0.014940875
EPHB6	404.1111111	350.3888889	2.70995981	0.01494708
WBP4	-255.6666667	767.0555556	-2.703252253	0.015157372
GPR132	271.7777778	331.6666667	2.702923223	0.015167761
NGRN	-2270.777778	7907.5	-2.698662154	0.015302911
TACC1	1618.111111	3141.166667	2.698460498	0.015309336

PPCDC	-311.7777778	535.8888889	-2.697823839	0.015329636
CXCL9	9181.333333	5748.222222	2.689639823	0.015592899
EGFL8	-250.3333333	229.5	-2.68821747	0.015639093
LYPLA1	-1754.666667	5261.222222	-2.686726234	0.015687665
HERPUD2	410.1111111	1833.611111	2.686450754	0.015696653
ATG5	-993.2222222	2220.277778	-2.684672283	0.015754802
JAK2	996.8888889	1409.555556	2.6827518	0.015817825
KIAA2013	1077.777778	3883.111111	2.677591485	0.015988367
DCUN1D4	-741.6666667	2110.388889	-2.677499871	0.015991411
BAT4	-351.2222222	989.2777778	-2.670529444	0.016224615
TBP	-304.3333333	886.7222222	-2.667780896	0.016317463
SEC23B	2708.777778	7723.833333	2.667509813	0.016326647
IL4R	1557.333333	2786.666667	2.665280496	0.01640237
ROGDI	-1580.333333	3743.833333	-2.665028321	0.016410956
VPS26B	1298.666667	3652.333333	2.663366618	0.016467645
ARL6IP4	-2495.555556	7321.333333	-2.661670761	0.016525693
NCAPD3	914.8888889	2395.777778	2.660317972	0.016572138
HNRNPA3	-5002.222222	15686.22222	-2.658766954	0.016625542
MVK	-409.3333333	1107	-2.652983057	0.016826147
RAPGEF1	1754	4189.666667	2.652189534	0.016853849
C19orf62	-1188.111111	3534.277778	-2.647606714	0.01701469
C5orf54	-177.1111111	501	-2.646766583	0.017044335

TECR	-2410.777778	5862.277778	-2.645290915	0.017096524
COQ5	-757.6666667	2084.055556	-2.642176645	0.017207167
C19orf21	-3300.222222	3802	-2.640042571	0.01728338
BIN1	1090.777778	1215.722222	2.638688236	0.017331914
STIM2	360.4444444	1246.666667	2.638392624	0.017342525
NDUFB7	-1566.888889	3399.666667	-2.636453535	0.017412282
ADCK1	-285.6666667	563.1666667	-2.636201479	0.017421369
C12orf52	-512.4444444	1604	-2.634871771	0.017469383
SH3TC1	565.1111111	1074.777778	2.633569498	0.017516528
GSTM4	-848.4444444	1305.111111	-2.631821595	0.017579998
RMND5B	-951.8888889	2575.722222	-2.630725937	0.017619895
SHC4	459	302.0555556	2.626834078	0.017762313
C6orf125	-1367.111111	2929.222222	-2.625013634	0.017829306
TMC8	700.3333333	880.2777778	2.623365916	0.017890151
C10orf76	-504.3333333	2142.611111	-2.62050873	0.017996128
STAT5B	1614.111111	4916.833333	2.612345986	0.018302205
C8orf84	4554.222222	2720.555556	2.61128714	0.01834227
TMEM231	352.1111111	638.1666667	2.609897633	0.018394974
C6orf136	-366.4444444	742.3333333	-2.608028983	0.01846608
C10orf26	-1685.111111	5296.444444	-2.607415362	0.018489486
PCDHA1	-473	285.6111111	-2.606814625	0.018512429
SYT7	7620.666667	6773.222222	2.603385104	0.018643925

C16orf48	244.7777778	847.7222222	2.602131046	0.01869223
SLTM	-1535.222222	5864.055556	-2.599308107	0.018801404
SAMD4B	1412.555556	4384.944444	2.599082459	0.018810157
CLIP3	612.7777778	991.9444444	2.598263285	0.018841965
CADM4	-1154.777778	2375.055556	-2.598149697	0.01884638
TNIP1	1868.333333	6690.5	2.597256669	0.018881122
SOD2	7603.333333	10298	2.596490641	0.018910973
ATM	1086.777778	2890.944444	2.595996004	0.018930271
PCDH7	981	1247.388889	2.595034926	0.018967822
CDH2	324.3333333	325.0555556	2.590650891	0.019140014
DPY30	-793.7777778	2114.111111	-2.586790698	0.019292863
SAMD10	379.1111111	796.2222222	2.584076999	0.01940101
ETV4	610.3333333	555.5	2.581737345	0.019494714
PDLIM4	917.7777778	1166	2.580229893	0.019555315
SSNA1	-1208.666667	3261.222222	-2.579788403	0.019573098
APOBEC3G	681.6666667	777.5	2.578784109	0.019613606
PECI	-1149.888889	3110.166667	-2.577125334	0.019680688
TRIM56	878	1320.888889	2.572489011	0.019869344
ZNF830	-442.2222222	1097.333333	-2.567023193	0.020093961
PQBP1	-676.5555556	2561.944444	-2.564131265	0.020213779
ZBTB44	1379	4165.277778	2.563801065	0.020227503
KLHL21	821.3333333	2471	2.562937032	0.020263456

PSTPIP1	306.1111111	287.5	2.561832794	0.020309493
TMEM14C	-2137.111111	6190.111111	-2.560874165	0.02034954
LOC222699	-192.5555556	250.2777778	-2.557539786	0.02048942
VARS2	-819.2222222	2498.388889	-2.55601302	0.020553773
HEXIM2	-341.5555556	554.4444444	-2.555736759	0.020565438
ATAD3C	-416.5555556	557.9444444	-2.555019116	0.020595769
SDF2	-919.5555556	2962.666667	-2.549025891	0.020850739
EXOC2	-2634.111111	4055.5	-2.547639464	0.020910147
LOC646214	-228	425.6666667	-2.547480263	0.020916979
NUDT14	-636.4444444	1184.222222	-2.545727624	0.020992333
CYB5R1	-1763.222222	5510.611111	-2.542373807	0.021137248
PLXNA1	3025	4765.5	2.541840995	0.021160357
ERH	-3272.888889	7206.444444	-2.540313039	0.021226761
TRIB2	1322.555556	2661.611111	2.538767454	0.021294133
LYPD3	-2388.888889	2707.666667	-2.537675122	0.02134187
TPD52	-11004.22222	18744.11111	-2.536163702	0.021408089
ANO6	1121.666667	5261.388889	2.531280081	0.021623388
TCEAL3	-2553.888889	3105.055556	-2.528266653	0.02175726
DNAJC5	2220.777778	6983.055556	2.524920113	0.021906852
CSNK2B	-3086.444444	7994.444444	-2.517742661	0.022230981
NEDD4L	-2912.666667	4757	-2.516911814	0.022268794
FADS3	666.5555556	1290.944444	2.515514374	0.02233253

IPO8	-694	3474.444444	-2.512723146	0.022460354
C6orf70	-517.5555556	1240.333333	-2.509368894	0.022614878
DIABLO	-778.8888889	2636.666667	-2.508989129	0.022632436
ASAM	399.6666667	590.2777778	2.508709261	0.022645384
MATK	231.6666667	198.6111111	2.508309011	0.022663913
STAT4	152.2222222	152.1111111	2.505684511	0.02278577
OVOL2	-291.2222222	715.3888889	-2.504996849	0.022817801
QPCT	1228.555556	1023.722222	2.500352705	0.02303524
C3	18230.66667	22113.88889	2.49741723	0.023173689
BNIP1	-229.3333333	602.4444444	-2.496252095	0.02322886
MAPK13	-1415	4208.277778	-2.496032342	0.023239279
EGLN1	933.5555556	3520.555556	2.493311345	0.023368661
TSC22D1	-4355.333333	11802.11111	-2.491763533	0.023442562
ORAI2	2200.777778	3193.944444	2.490402003	0.023507751
HIF1A	3556.666667	6278.888889	2.490039843	0.02352512
OMD	-401	479.2777778	-2.489928261	0.023530474
NDUFA12	-993.4444444	2019.833333	-2.488819824	0.023583721
ANAPC16	-1757.111111	6013.111111	-2.488724919	0.023588285
SRD5A3	-1103.777778	2487.888889	-2.4883642	0.023605641
HR	2077.777778	1584.444444	2.488031949	0.023621638
ERI1	397.1111111	971.5555556	2.487139847	0.02366464
RNF145	1220.777778	2075.5	2.484843134	0.023775691

COQ3	-250.1111111	403.2777778	-2.484464334	0.023794054
WDFY1	1837.333333	3559.888889	2.484315305	0.023801282
FKTN	489.7777778	1583.888889	2.483061914	0.023862155
C19orf53	-1510.333333	3851.5	-2.482956654	0.023867274
TRIM29	3163	2297.5	2.479389076	0.024041382
NLK	-338.7777778	1599.944444	-2.476674855	0.024174646
PFDN6	-722.6666667	1667.111111	-2.47598421	0.024208667
MTG1	-588.1111111	1328.833333	-2.475455614	0.024234736
ZCCHC8	-428.2222222	1524	-2.474836904	0.024265283
CSF1R	1513.333333	2882.666667	2.474339387	0.024289873
RPP40	-240.4444444	410.8888889	-2.473933488	0.024309952
LYRM2	-914.7777778	2544.833333	-2.473669317	0.024323028
C6orf203	-496.4444444	793.7777778	-2.472908659	0.024360717
DENR	-945.6666667	4771.166667	-2.469258672	0.024542337
SERF1A	-842.2222222	1839.777778	-2.467053735	0.024652671
AKAP8L	-1032.555556	2593.055556	-2.466567021	0.024677089
N6AMT2	-213.6666667	475.3888889	-2.457945111	0.025113443
ZFP64	334	1114.777778	2.454180978	0.025306219
AHDC1	1732.222222	3103.333333	2.453564325	0.025337932
GSTZ1	-797.7777778	1382	-2.451367318	0.025451227
COMMD4	-1521.111111	3057.444444	-2.450485739	0.025496823
LOC440905	-722.6666667	570	-2.449174247	0.025564796

KIAA1598	-2135	5989.166667	-2.444604107	0.025802997
ADCK5	-244.6666667	604.5555556	-2.444061565	0.025831413
C17orf90	-549.5555556	1489.111111	-2.442477029	0.025914574
EVC	683.8888889	1568.5	2.441927291	0.025943485
C12orf76	-244	491.4444444	-2.439496269	0.026071698
STX8	-368.2222222	1013.333333	-2.438349971	0.02613236
ATP2B4	4465.111111	8913	2.435728063	0.026271613
TSPAN13	-9756.666667	16055.55556	-2.434012026	0.026363133
PPP1R11	-1767.111111	5165.888889	-2.431437693	0.026500989
TBC1D5	1016.666667	3743	2.430830389	0.026533609
ACADS	-540	1295.777778	-2.430718248	0.026539637
SF3B2	2726.111111	12630.72222	2.430461689	0.026553431
PCDHB4	-228.6666667	241.1111111	-2.430380768	0.026557784
ARHGAP26	1181.666667	1358.055556	2.42901245	0.026631482
PLOD2	4652.555556	5103.611111	2.427067984	0.026736543
ADI1	-1971.111111	4078.222222	-2.420968486	0.027068638
SURF4	3607.666667	13544.61111	2.41994072	0.027124977
CYBASC3	851.1111111	2801.777778	2.419895911	0.027127436
WDSUB1	-278.3333333	712.2777778	-2.419495094	0.027149439
LOC642826	-133.6666667	219.7222222	-2.419197683	0.027165777
PDZD11	-1148.111111	2872.5	-2.418914482	0.027181342
C19orf42	-1645.888889	4300.055556	-2.418518512	0.02720312

SMARCD3	-842.3333333	1121.833333	-2.417502713	0.027259062
QSER1	1249.222222	3735.277778	2.417449902	0.027261973
LASS4	-2423.777778	3514.111111	-2.416065771	0.027338382
LIMS1	492.3333333	937.7222222	2.414516525	0.027424145
DENND5A	2007.666667	4688.722222	2.414182681	0.027442658
PPP3CA	2862.111111	4649.5	2.412774397	0.027520887
ARFIP1	-826.4444444	3086.777778	-2.410988129	0.027620413
GNA15	585.2222222	954.9444444	2.408703202	0.027748216
BIRC2	1354.111111	3440.944444	2.40698697	0.027844575
OBFC1	-459.6666667	1746.166667	-2.405108583	0.0279504
ATG2A	1148.666667	2821.111111	2.401655997	0.028145896
HTRA2	-325.8888889	1551.5	-2.399892886	0.028246224
IER3IP1	-1994.222222	4490.222222	-2.399147911	0.028288717
RBCK1	2932.666667	7263.777778	2.398600213	0.028319996
TMEM39A	453.1111111	1567.444444	2.398204376	0.028342622
CCDC8	928.3333333	990.3888889	2.396513441	0.028439467
SPRN	-170.6666667	363.6666667	-2.395238206	0.028512709
KIF22	-1881.333333	4556	-2.3939805	0.028585118
MRPS11	-702.8888889	1687.333333	-2.393653127	0.028603994
TMEM116	-340.5555556	713.9444444	-2.391925403	0.028703806
ATRN	1531.777778	6491	2.391517581	0.028727414
SEPP1	-9362.666667	14633.66667	-2.390484587	0.028787293

VPS29	-1035.333333	3179.333333	-2.389589858	0.028839252
MFHAS1	381.5555556	514.1111111	2.389295094	0.028856388
C9orf119	-361.1111111	964.3333333	-2.388908293	0.028878891
FBXO31	533.3333333	1429.444444	2.387372916	0.028968374
NDUFB8	-1716.777778	3427.722222	-2.385447804	0.029080938
FN3K	-569.7777778	1258.222222	-2.384724588	0.029123332
DAD1	-2660.666667	8445.222222	-2.383407478	0.029200687
PDPR	2227.333333	2569.666667	2.381332315	0.029322955
ST5	1797.222222	3096.833333	2.380173404	0.029391446
TMEM141	-1538.888889	3460.333333	-2.378815854	0.029471868
TNFAIP8	384.6666667	995.3333333	2.376634459	0.029601526
RPN2	10444	26958.77778	2.375574725	0.029664708
C20orf194	458.8888889	1460.888889	2.373413188	0.029793971
C12orf43	-191	662.0555556	-2.372299907	0.029860752
NAB1	839.1111111	2448.111111	2.372240695	0.029864308
NFIL3	697	1253.833333	2.372162832	0.029868985
DIAPH1	2520.666667	8875.222222	2.371964557	0.029880896
C6orf162	-222.4444444	287.6666667	-2.365077373	0.030297422
CEBPB	2293	3704.722222	2.363608216	0.030386975
FMNL1	976.8888889	1639.555556	2.36181628	0.030496539
IDO1	1356.666667	837.5555556	2.360222987	0.030594267
KLHDC8B	-505.5555556	1246.555556	-2.358981423	0.030670624

UBL7	-532.3333333	2122.611111	-2.357048229	0.030789873
LONRF1	561.8888889	1144.833333	2.356353139	0.030832855
C22orf39	-336.7777778	1128.055556	-2.354108363	0.030972049
BIRC3	1534.777778	1351.166667	2.353697607	0.030997583
TM9SF4	1746.222222	6484.666667	2.352125549	0.031095488
PATL1	1216.444444	3287.222222	2.35052772	0.031195294
TSPAN12	-619.2222222	876.1666667	-2.350278599	0.031210882
BRWD1	-1281.888889	5298.166667	-2.350163348	0.031218096
ATP5I	-1589.333333	3554.444444	-2.34753458	0.031383064
GSTM3	-8557.888889	7344.166667	-2.345610999	0.031504294
ORAOV1	-774.6666667	1042.333333	-2.345106351	0.03153617
SLC25A37	972.2222222	1328.555556	2.344889773	0.03154986
FAT1	9922	9726	2.342768298	0.03168425
KRT23	2188.555556	1755.166667	2.342477448	0.031702717
NOB1	641	2193.611111	2.341525305	0.031763239
TIMM13	-1586.111111	3587.722222	-2.34085244	0.031806075
HSF2	-260.5555556	897.1666667	-2.34061345	0.031821302
AMOT	1861.111111	3070.333333	2.339190411	0.031912114
GLG1	6130.444444	11543.55556	2.339117981	0.031916742
ZAP70	341.3333333	302.2222222	2.33628206	0.032098463
C10orf140	150.1111111	116.6111111	2.336147088	0.032107136
ZNF165	-266.4444444	370.8888889	-2.333905128	0.032251517

APOL1	4878.444444	4981.777778	2.333768025	0.032260366
USP45	-272.3333333	626.6111111	-2.333504473	0.032277383
FAM178A	-577.7777778	2598.888889	-2.333346266	0.032287602
DST	8613.777778	11266.88889	2.333274318	0.03229225
TTPAL	619.2222222	1733.611111	2.331613913	0.032399696
C17orf28	-5033.888889	8893.5	-2.331093368	0.03243345
SLC27A4	-1034.333333	2767.277778	-2.328503228	0.03260189
NFATC3	1344.444444	2359.222222	2.327526651	0.03266561
CNFN	-161.6666667	262.9444444	-2.325541223	0.032795514
GPX4	-5004.888889	13033.22222	-2.325343403	0.032808483
EIF2B1	-864.6666667	3359.555556	-2.325183483	0.032818971
BRF1	-1021.333333	2939.666667	-2.32393316	0.03290108
DPYD	841.2222222	1323.055556	2.322588668	0.032989587
FKBP5	4177.444444	5675.166667	2.32103947	0.033091845
CHST3	1215.222222	1402.5	2.32051768	0.033126353
AP2A1	3201.666667	9545.611111	2.319241912	0.033210867
TERF2	562.6666667	1703.444444	2.315166931	0.033482163
TREM2	-591	1087.611111	-2.314748148	0.033510161
C9orf25	491.6666667	1179.611111	2.312003105	0.033694222
NRF1	-218.8888889	1175.666667	-2.310870884	0.033770414
TBC1D10C	316.5555556	374.5	2.309386133	0.033870572
NUDT4	-1202.222222	2478.666667	-2.308871247	0.03390537

SSC5D	1557.444444	1798.388889	2.306340068	0.034076921
PCDHGA10	-232	198.5555556	-2.305076364	0.034162871
GIPC1	-2154.888889	7012	-2.304950053	0.034171473
RPS27L	-1781.333333	3452.222222	-2.303931833	0.034240889
THBD	-959.1111111	1711.333333	-2.30377599	0.034251525
HOXC9	-296.6666667	473.7777778	-2.300830067	0.034453161
DDOST	3608.444444	12823.55556	2.297749032	0.034665226
PISD	-1589.111111	2264.222222	-2.295346948	0.034831401
SMARCE1	-6656.222222	12518.55556	-2.294511669	0.034889359
C15orf57	-505.7777778	1202.222222	-2.292694055	0.035015789
HDHD2	-818.8888889	2575.777778	-2.292034032	0.035061804
SLC39A14	3155	4154.722222	2.289893877	0.035211399
DTNBP1	-305.4444444	1047.166667	-2.289127478	0.035265113
C6orf47	-675.3333333	1953	-2.287696914	0.035365581
PLTP	2273.888889	2847.388889	2.287469261	0.035381593
CXCL16	1934.777778	3805.611111	2.285087317	0.035549537
POLR2H	-1909.222222	3312.166667	-2.282202824	0.035753907
POLR2L	-2740.888889	5348.222222	-2.282072398	0.035763174
ACOX3	661.5555556	1526.444444	2.281094335	0.035832735
PLAGL2	655.2222222	1753.166667	2.279729145	0.035930039
RAP1GAP2	851.7777778	1265.777778	2.279447584	0.035950138
MARK2	2530.666667	5554.666667	2.278814608	0.03599536

DDX41	-665.7777778	3312.444444	-2.277977835	0.036055224
MRPS9	-440	1382.111111	-2.277287321	0.036104694
ECE1	9278.444444	21202.44444	2.276779916	0.036141085
C9orf89	-574.2222222	1402.666667	-2.276583314	0.036155195
CXorf40A	-763	1849.944444	-2.276228588	0.036180666
HMGN3	-2239.222222	3160.388889	-2.274203784	0.036326374
SIPA1	864.5555556	2224.611111	2.274163843	0.036329254
ADSSL1	-218.1111111	369.7222222	-2.273165396	0.03640131
ANKH	3419.666667	5982.833333	2.272754497	0.036431002
CCNC	-1497.888889	3688.944444	-2.272415206	0.036455537
FAH	-3598.333333	3660.833333	-2.272121428	0.036476793
PGM5	-201.8888889	242.7222222	-2.271364311	0.036531626
NOL7	-646.8888889	2212.111111	-2.269822888	0.036643499
HSD17B2	133.6666667	82.83333333	2.269624659	0.036657909
C22orf9	1677.222222	5529.722222	2.269309264	0.036680847
POLR3D	307.1111111	869.3333333	2.268868245	0.036712944
CD247	194	201.2222222	2.267237593	0.036831847
GNPTAB	1201.333333	3534.333333	2.267177408	0.036836243
KAZALD1	-415.4444444	436.0555556	-2.267016686	0.036847983
SERPINB8	252.7777778	603.8333333	2.266530675	0.036883505
ST6GAL1	2763	4490.166667	2.264017618	0.037067691
C20orf177	280.4444444	642	2.263613584	0.037097383

SLC25A35	-440.4444444	722.1111111	-2.263141035	0.037132138
SPR	-1280.222222	3436.777778	-2.262592999	0.037172482
ZSCAN16	-314.7777778	511.0555556	-2.260561332	0.037322402
MBNL3	110.3333333	101.2777778	2.25972578	0.037384221
C2orf7	-384.1111111	766.5	-2.259621652	0.037391932
MRPL52	-625	1464.055556	-2.259426493	0.037406387
BLNK	-547.2222222	950.9444444	-2.259185422	0.037424251
CDC42BPG	1356	2215.777778	2.259125158	0.037428718
SMEK1	-1427.444444	5018.611111	-2.258782184	0.037454148
GBGT1	218.1111111	343.0555556	2.255263834	0.037715954
ZNF346	-224.7777778	848.9444444	-2.25283635	0.037897575
TMX4	1477	2739.5	2.252445441	0.037926898
TNPO1	1725.888889	8180.944444	2.251973083	0.037962358
BCAS2	-803	2131.277778	-2.251657503	0.037986066
FBXO32	1269.333333	2097.333333	2.251237004	0.038017678
ISCU	-830.6666667	3417.777778	-2.249904897	0.038117982
TRIM27	-1499	4581.944444	-2.249432753	0.038153592
SEC63	-2202.888889	7944.777778	-2.249406123	0.038155602
PTPN7	360.5555556	448.9444444	2.248932384	0.038191365
CABLES2	545.1111111	983.8888889	2.248183016	0.038247998
TSSC4	-491.1111111	1925.666667	-2.246114465	0.038404735
IRF6	1616.666667	3760.222222	2.245695945	0.038436519

POLL	-457.4444444	1379.388889	-2.245012108	0.038488504
ITPR3	1745.555556	5326	2.243633193	0.038593528
GBP5	2791.444444	1774.5	2.242006033	0.0387178
B3GNT7	203	296.0555556	2.240209989	0.038855401
VPS33B	-369.4444444	924.2777778	-2.239072623	0.038942773
ATP2B1	-1183.222222	3299.055556	-2.237991041	0.039026028
SYNC	-953	1017.944444	-2.237110352	0.03909394
FAM47E	-230.7777778	325.5	-2.236938884	0.039107176
TRAK2	-2157.333333	4186.777778	-2.236909274	0.039109461
ANAPC5	-1575.111111	7534.444444	-2.236900089	0.039110171
U2AF1	-1865.777778	4205.444444	-2.236315024	0.039155365
C14orf119	-805.8888889	3020.5	-2.234276044	0.039313246
ACAP1	310.5555556	344.0555556	2.234172108	0.03932131
ZFAND6	-1682	4700.555556	-2.233989877	0.039335452
DHRS11	-257	618.1666667	-2.2329998	0.039412367
CDK19	-734.7777778	2089.277778	-2.232346049	0.039463231
MGA	803.8888889	2619.277778	2.232063984	0.039485195
SDC3	5705.888889	8513.944444	2.231353558	0.039540566
TLN1	7089.777778	17557.44444	2.228919297	0.039730838
PTPRK	3254.666667	6101	2.228545165	0.039760157
KLF2	-1009.111111	1543.666667	-2.22703906	0.039878385
LRRC1	-1031.333333	2255.111111	-2.22683575	0.039894369

COMMD9	-643.1111111	2006.666667	-2.22677919	0.039898817
NKG7	484.4444444	374.3333333	2.22623851	0.039941359
MYO9B	1417.777778	5254	2.225754092	0.03997951
SBNO2	2294.555556	6669.277778	2.225489732	0.040000344
CYTIP	339.7777778	530.6666667	2.225316409	0.04001401
CBLB	333.4444444	1180.277778	2.224114162	0.040108915
MGEA5	-3151.222222	11573.16667	-2.222627992	0.040226522
CD44	14279.88889	24905.5	2.221822184	0.040290422
GZMB	339.7777778	197.5555556	2.221642808	0.040304659
EGFL7	-1013.888889	1345.388889	-2.220747517	0.040375788
ZSCAN12P1	-96.11111111	141.9444444	-2.218860129	0.040526117
AGAP4	-363.5555556	602.2222222	-2.218847654	0.040527112
RELB	1236.444444	1755.222222	2.21848943	0.040555704
GLS	1583.222222	3210.611111	2.218408671	0.040562152
RDBP	-1551.888889	3937.611111	-2.217130656	0.040664323
CRIM1	6413	7868.388889	2.215125646	0.040825091
TOR3A	1074	3650.555556	2.213268101	0.04097456
IL2RA	146.3333333	110.8333333	2.213218617	0.040978549
OSBPL3	691.1111111	1223.222222	2.212098688	0.041068916
CCDC12	-372.6666667	1360.888889	-2.211002076	0.041157581
CD7	298.8888889	302.1111111	2.210839672	0.041170727
SH3GLB2	-2434.222222	5813.555556	-2.210271622	0.041216739

CIITA	1748.222222	1384.222222	2.207715666	0.041424357
ZDHHC5	1820	7540.888889	2.207658498	0.041429012
BICD2	488.7777778	2336.277778	2.206970518	0.041485067
COMT	-1689.222222	4863.5	-2.204085638	0.041720881
NLRC5	3190.666667	2544.222222	2.204072841	0.04172193
ITGAL	1453.666667	1757.833333	2.20376222	0.041747395
PRICKLE3	175	621.7222222	2.203652114	0.041756425
ATP5F1	-2443.555556	8191.444444	-2.203124084	0.041799755
INO80C	-343.7777778	629.5555556	-2.202843354	0.041822808
NPTXR	1309	1712.833333	2.202545452	0.041847284
HSPA5	14051.66667	46453.61111	2.201675528	0.041918835
EDEM2	822.1111111	1983.055556	2.199535025	0.042095371
SDS	-433.6666667	554.2777778	-2.199456599	0.042101852
LOC550112	-282.4444444	773.2222222	-2.198145315	0.042210352
ZNF239	-395.3333333	781.1111111	-2.197850956	0.042234744
KCTD17	245.6666667	674.2777778	2.197284742	0.0422817
BTG3	773.5555556	1063.222222	2.196230786	0.042369231
CAMK1	-193.2222222	579.9444444	-2.195932094	0.042394068
NDUFS4	-904.6666667	1876.666667	-2.195623039	0.04241978
ACTR1A	-2054.888889	7227.222222	-2.194703195	0.042496394
STAT5A	3365.333333	4193.333333	2.194651354	0.042500716
SIRT5	-448.6666667	1176.555556	-2.194110021	0.042545867

PRPF38B	-820.5555556	3178.833333	-2.193918129	0.042561883
INPP5D	799.5555556	1233.222222	2.193850822	0.042567502
FRAT1	-263.5555556	599.7777778	-2.193271892	0.042615861
TGFB2	1258.888889	1214.333333	2.193047256	0.042634639
NDUFB4	-2172	6024.888889	-2.192854417	0.042650765
RAP1GDS1	-3595.777778	4849.666667	-2.190402943	0.042856257
NCK1	511.2222222	1657.611111	2.190281018	0.042866501
CXCR3	204.7777778	214.0555556	2.189545833	0.042928318
FNDC3B	3436.444444	4911.777778	2.189112307	0.042964809
GBP1	8663.111111	5871	2.188656106	0.04300324
ZBTB9	-331.3333333	1095.777778	-2.187635765	0.043089309
FOXP3	233.3333333	238.2222222	2.184545906	0.043350915
POLR2E	-1401.666667	5724.944444	-2.183734496	0.043419856
MSX2	-489.6666667	599.0555556	-2.180782924	0.043671488
HCK	941.1111111	1043.111111	2.179099607	0.043815596
TRIM25	1264.222222	3841.666667	2.178575194	0.043860581
ZFP91	1590.777778	5741.388889	2.177313087	0.043969019
SERF2	-15586.77778	26203.38889	-2.177170491	0.043981286
LAMA5	6246.555556	11400.05556	2.176641691	0.044026805
TSPAN5	-487.2222222	398.0555556	-2.176625366	0.044028211
CCDC99	-249.6666667	732.5	-2.176378222	0.0440495
LOC338799	-484.1111111	739.7222222	-2.176146743	0.044069448

DOCK8	1097.333333	2374.444444	2.174877091	0.044179014
NAA35	473.8888889	1447.611111	2.173115417	0.044331453
LAMC2	13638.66667	9085.666667	2.173100133	0.044332778
LAMC1	8384.777778	14636.72222	2.171250765	0.04449333
ARSD	4145.333333	9371.333333	2.167656413	0.044806903
LOC202781	-443.8888889	738.8333333	-2.167502981	0.044820334
C6orf182	-219.4444444	468.3888889	-2.167139982	0.044852123
C19orf28	830.8888889	3907.555556	2.16693548	0.044870042
CELSR2	9981.333333	13654.66667	2.166910843	0.044872201
SETX	1527.222222	5422.722222	2.165965213	0.044955146
FAHD2A	-211.1111111	631.1111111	-2.165515059	0.04499468
HAS3	648.4444444	492.7777778	2.164217257	0.045108837
MST4	-739.1111111	1281.111111	-2.163113097	0.045206171
OSGEP	-284.5555556	1133.388889	-2.162885265	0.045226278
HYDIN	371.6666667	412.3888889	2.162398107	0.045269301
NLRC3	222.8888889	344	2.161612075	0.045338798
PRR24	-194.1111111	638.7222222	-2.161077629	0.045386106
NHP2	-1684.111111	4424.388889	-2.160918371	0.045400212
CYP4Z2P	-503.2222222	416.2777778	-2.16089946	0.045401888
JPH2	270.7777778	244.8333333	2.160885371	0.045403136
ATL3	782.4444444	1661.111111	2.15978278	0.045500913
STK19	-475.3333333	1141.444444	-2.157487243	0.045705101

PIK3CD	442.8888889	825.6666667	2.156696908	0.045775596
FAAH2	-324.4444444	849.8888889	-2.156346292	0.045806902
GLIS3	227.6666667	439.7222222	2.155792431	0.045856395
CHI3L1	9554.222222	5991.111111	2.154894716	0.045936718
KRT17	16356.22222	10472	2.154872125	0.045938741
HLA-F	2946.444444	3765.777778	2.154182046	0.046000577
ARX	-134.6666667	75.33333333	-2.154120278	0.046006115
SPRYD4	-311.8888889	899.1666667	-2.15277965	0.046126476
ICA1	-1332.666667	3082.444444	-2.150850925	0.046300143
TUT1	421.6666667	1677.5	2.150587795	0.046323883
STMN3	3886.666667	4417.777778	2.14955972	0.046416742
HSD17B7P2	-118.2222222	169.3333333	-2.14940509	0.046430723
BTN3A3	888.1111111	1591.388889	2.148519308	0.046510889
ZNF334	174.2222222	235.2222222	2.146680177	0.046677741
LZTS2	-2170	6570.666667	-2.146221793	0.046719412
SAP30BP	-784.1111111	2779.277778	-2.145567889	0.046778917
NAGK	-1299.333333	3473.777778	-2.145519527	0.046783321
ITGB8	1498.444444	1733.222222	2.144208445	0.046902849
DDAH2	-2905.777778	5736.111111	-2.143531778	0.046964649
C10orf114	187	166.7222222	2.142550208	0.047054427
ZMYM5	-236.8888889	879.7777778	-2.142511172	0.047058001
TCF7	457.6666667	838.2777778	2.141718885	0.047130587

MRPL43	-1316.888889	3092.666667	-2.141194596	0.047178676
TBL1X	1562.333333	3394.944444	2.140558196	0.04723711
AGPAT1	-1259.222222	4985.722222	-2.139739633	0.047312366
ZNF271	-631	2220.166667	-2.138896587	0.047389989
TK2	532.6666667	2095.555556	2.137231664	0.047543627
CSNK2A1P	417.6666667	1448.277778	2.136299134	0.04762988
ARHGAP5	1822.555556	5162.5	2.136096562	0.047648636
LRRC8A	864.4444444	3238.444444	2.134909461	0.047758682
ZNF69	-137.1111111	233.2222222	-2.134337742	0.047811764
KITLG	-4314.888889	4482	-2.133523923	0.047887418
AMIGO2	4348.888889	3439.444444	2.133153549	0.047921884
ZNF229	272	405.2222222	2.130835133	0.048138151
VTI1A	-423	1272.5	-2.13040573	0.048178305
RILPL1	-187.6666667	657.9444444	-2.130197242	0.048197812
POP5	-302.1111111	884.9444444	-2.129530712	0.048260223
NHLRC1	-260.4444444	425.3333333	-2.128790936	0.04832958
MRPL14	-1278.888889	2488.666667	-2.126700108	0.048526095
BAG3	-3344.666667	4905.333333	-2.126500121	0.04854493
CINP	-499.8888889	1278.611111	-2.126459376	0.048548768
USE1	-320.3333333	753.0555556	-2.126358674	0.048558256
RRP9	-245.7777778	1068.777778	-2.12590564	0.048600958
CPT1B	-855.5555556	996.3333333	-2.124071287	0.048774214

MSN	14314.66667	20597.44444	2.123610236	0.04881785
SNX3	-2143.222222	7501.5	-2.122917135	0.048883514
ZNF498	221.1111111	961.7777778	2.12173094	0.048996082
SUGT1	-544.8888889	1786	-2.120059682	0.049155085
PSMB1	-3234.666667	8716.666667	-2.119809815	0.049178897
AK1	-608.2222222	1696.888889	-2.119551015	0.049203572
SLC29A1	-1884.666667	6337.888889	-2.119234026	0.049233811
NDP	-473.8888889	318.2777778	-2.117611339	0.04938887
YES1	1888.888889	4885.888889	2.117310402	0.049417676
C1orf58	509.7777778	1362.111111	2.117304337	0.049418257
VAV1	322.1111111	568.3888889	2.116426846	0.049502339
MTERFD3	-554.8888889	1103.666667	-2.116056013	0.049537912
MOB2	-330.6666667	1339.333333	-2.115059965	0.049633575
DOC2B	171	114.7222222	2.114783241	0.049660183
C4orf42	-596.5555556	1571.611111	-2.114422595	0.049694879
SP110	1069.666667	2208.611111	2.113659221	0.049768393
ELMOD3	-301.3333333	932.6666667	-2.113508701	0.0497829
PLA2G16	-1740.666667	3394.111111	-2.11207383	0.049921386
CDC40	-502.5555556	2008.944444	-2.111826368	0.049945306
SMN2	-879.2222222	3046.611111	-2.111366403	0.049989793

Table S3. Results of differential analysis of high-risk and low-risk TNBC patients.

Gene	logFC	AveExpr	P.Value	adj.P.Val	B	change

NR2E1	1.564877273	2.231702273	2.13E-06	0.043745898	1.282089609	UP
COL22A1	2.317306818	6.606482955	0.000132404	0.662477298	-0.845043663	UP
RAPSN	1.209615909	1.583119318	0.000255038	0.662477298	-1.186553202	UP
ARHGEF4	1.132743182	7.409710227	0.000282226	0.662477298	-1.239368929	UP
OSBPL6	-1.097904545	6.006172727	0.000290419	0.662477298	-1.254290735	DOWN
PKP1	2.093038636	9.663367045	0.000649896	0.802979349	-1.674519891	UP
KRT16	2.200495455	9.830222727	0.0007106	0.802979349	-1.721113284	UP
CDKN2A	1.9127	8.254131818	0.000747468	0.802979349	-1.747504629	UP
LOC285629	1.154556818	2.430803409	0.000755669	0.802979349	-1.753198146	UP
SCT	1.009370455	2.049998864	0.001016283	0.802979349	-1.907768253	UP
CDH2	1.186877273	6.525929545	0.001041313	0.802979349	-1.920457555	UP
PIP5K1B	-1.399095455	5.754759091	0.00110968	0.802979349	-1.953619079	DOWN
KANK4	1.395497727	8.104994318	0.00127739	0.802979349	-2.027001953	UP
TFCP2L1	1.26365	9.936806818	0.00134155	0.802979349	-2.052547202	UP
RBPMS2	-1.028197727	5.985405682	0.001486765	0.802979349	-2.106108928	DOWN
UGT2B28	-1.594320455	1.0248875	0.001497062	0.802979349	-2.109705143	DOWN
OCA2	1.773765909	3.907130682	0.001586715	0.802979349	-2.140007399	UP
DUSP9	1.283161364	5.489807955	0.002194985	0.802979349	-2.308953626	UP
GJB3	1.518377273	6.517275	0.002248029	0.802979349	-2.321375679	UP
S100A2	1.847788636	7.6385375	0.00226183	0.802979349	-2.324559184	UP
EDAR	1.403609091	3.175852273	0.002263173	0.802979349	-2.324868083	UP
C21orf128	1.005793182	1.912464773	0.00234633	0.802979349	-2.343636289	UP

SERPINB5	1.558170455	9.211728409	0.002406294	0.802979349	-2.356759631	UP
LRAT	1.112245455	5.621429545	0.002681867	0.802979349	-2.413124108	UP
LY6D	2.182075	5.627139773	0.002847109	0.802979349	-2.444190906	UP
KRT79	1.933861364	2.851653409	0.002992342	0.802979349	-2.470032977	UP
LOC339535	1.635702273	4.484435227	0.003114891	0.802979349	-2.490874847	UP
ANXA8L2	1.793638636	6.646201136	0.003133378	0.802979349	-2.493947107	UP
C6orf15	1.826993182	4.329273864	0.003438243	0.802979349	-2.542135983	UP
KRT17	1.942220455	11.42780114	0.003586378	0.802979349	-2.56401785	UP
RET	-1.140225	6.138019318	0.003697813	0.802979349	-2.579886601	DOWN
GDF15	-1.188836364	5.429252273	0.003731015	0.802979349	-2.584521563	DOWN
CCDC160	-1.208661364	4.042398864	0.004278657	0.802979349	-2.65549638	DOWN
KRT13	1.767675	2.556014773	0.004300352	0.802979349	-2.658115838	UP
SLC6A15	2.25505	4.606970455	0.004382296	0.802979349	-2.66789083	UP
LOC90246	-1.220697727	3.605271591	0.004646534	0.802979349	-2.698200106	DOWN
ANXA8	1.660256818	8.021044318	0.004839087	0.802979349	-2.719210372	UP
THSD7A	-1.019368182	4.991295455	0.00496257	0.802979349	-2.732244552	DOWN
WNT6	1.383336364	5.412686364	0.005241919	0.802979349	-2.760561467	UP
UGT8	1.311754545	7.457659091	0.005330467	0.802979349	-2.769219867	UP
FAT2	1.251409091	7.926693182	0.005441153	0.802979349	-2.779840978	UP
STEAP4	-1.204597727	8.264044318	0.005453799	0.802979349	-2.781040541	DOWN
KRT31	1.085236364	1.369679545	0.005480686	0.802979349	-2.783581598	UP
HGF	-1.054372727	4.559740909	0.005571397	0.802979349	-2.792062686	DOWN

IRX4	1.746495455	4.539772727	0.00596988	0.802979349	-2.82773686	UP
ARHGDIG	-1.565447727	2.491773864	0.006264269	0.802979349	-2.85257808	DOWN
L1CAM	1.307638636	6.956742045	0.006315445	0.802979349	-2.856775679	UP
S100A14	1.399297727	10.30676705	0.006325536	0.802979349	-2.8575993	UP
SLC1A6	1.862861364	3.083310227	0.006499831	0.802979349	-2.871619279	UP
SH3GL3	1.244136364	2.799409091	0.006609586	0.802979349	-2.880253914	UP
FERMT1	1.249815909	7.5178125	0.006659275	0.802979349	-2.884115408	UP
EGF	-1.392175	5.683269318	0.0068495	0.802979349	-2.898633689	DOWN
MYH14	1.143211364	10.69691023	0.007107575	0.802979349	-2.917691006	UP
APCDD1L	1.214625	6.202939773	0.007446857	0.802979349	-2.941705573	UP
WDR17	1.101156818	3.632051136	0.007706012	0.802979349	-2.959313563	UP
FOXQ1	1.194288636	4.931028409	0.00774145	0.802979349	-2.961674582	UP
SORCS2	1.052193182	6.036846591	0.007801322	0.802979349	-2.965638715	UP
MYO3B	1.385554545	4.041536364	0.007838571	0.802979349	-2.96808948	UP
PCDHA4	-1.171759091	3.701347727	0.007860544	0.802979349	-2.969529683	DOWN
UGT2B11	-1.738102273	1.742760227	0.007889707	0.802979349	-2.971434772	DOWN
CXorf49B	1.663875	3.478551136	0.008250538	0.802979349	-2.994433222	UP
KRT1	1.271356818	1.827101136	0.008361996	0.802979349	-3.001331455	UP
WNT11	1.134090909	5.742813636	0.008506892	0.802979349	-3.010161136	UP
SCNN1G	1.298370455	5.999055682	0.008599949	0.802979349	-3.015751746	UP
CYP4F8	-1.289265909	1.267035227	0.008616972	0.802979349	-3.01676776	DOWN
MAGEC2	-1.21145	0.976434091	0.008619462	0.802979349	-3.01691626	DOWN

C1orf61	1.034668182	3.203140909	0.008714776	0.802979349	-3.022566226	UP
MAGEB4	1.279731818	2.731570455	0.00932743	0.82826777	-3.057450782	UP
HGD	-1.524925	3.277551136	0.00965672	0.846474053	-3.075251373	DOWN
KLK6	1.889002273	7.992553409	0.010548221	0.856899309	-3.120512797	UP
DSC3	1.629270455	8.762217045	0.010620384	0.856899309	-3.124004756	UP
DNAJC22	-1.1159	5.258759091	0.010736353	0.856899309	-3.129566246	DOWN
KRT5	1.843513636	11.41240455	0.010768632	0.856899309	-3.131103405	UP
SDR42E1	1.064459091	7.578702273	0.010848796	0.859945132	-3.13490067	UP
ROS1	1.105577273	2.152668182	0.011025305	0.865983515	-3.143162045	UP
A2ML1	1.639713636	7.125604545	0.011221356	0.865983515	-3.152181852	UP
KLK10	1.645956818	7.674692045	0.012166531	0.871255393	-3.193530874	UP
TMEM45B	-1.287047727	4.376169318	0.012304392	0.871255393	-3.199287177	DOWN
DHRS2	-1.543406818	3.963714773	0.012587645	0.871255393	-3.210910872	DOWN
KLRG2	1.068125	5.8926375	0.012833091	0.871255393	-3.220769643	UP
TMC5	-1.721406818	5.753610227	0.013140754	0.871255393	-3.232859624	DOWN
SPINK8	-1.179838636	1.397105682	0.013583635	0.871255393	-3.249765842	DOWN
SPRR1B	1.578054545	1.947156818	0.013721036	0.871255393	-3.254896821	UP
PPP1R14C	1.083672727	8.429659091	0.013727008	0.871255393	-3.255118636	UP
CCNO	-1.099729545	4.547064773	0.01388003	0.871255393	-3.260769184	DOWN
SIGLEC14	1.026181818	5.094445455	0.01410821	0.871255393	-3.269078068	UP
HOXC10	-1.055161364	8.199880682	0.014503126	0.871255393	-3.283139608	DOWN
GRHL3	1.088468182	6.430129545	0.014523875	0.871255393	-3.283867561	UP

IVL	1.504813636	3.033945455	0.014544196	0.871255393	-3.28457949	UP
KRT9	1.161895455	2.731288636	0.014776142	0.871255393	-3.292634151	UP
GABBR2	1.491315909	5.072598864	0.015014456	0.871255393	-3.30077666	UP
MAGEA1	-1.150677273	1.118725	0.015039454	0.871255393	-3.301623095	DOWN
RAET1L	1.083920455	3.015928409	0.01512088	0.871255393	-3.304370302	UP
CALML5	1.954495455	8.459538636	0.015231599	0.871255393	-3.308081695	UP
KLK8	1.651929545	5.478980682	0.016128425	0.871255393	-3.337165905	UP
ACSM1	-1.020522727	2.991459091	0.016594229	0.871255393	-3.35162641	DOWN
C1QL2	1.172761364	3.768180682	0.016978812	0.871255393	-3.363256101	UP
GABRP	1.779027273	12.0759	0.017506097	0.871255393	-3.378770488	UP
ZP2	-1.185325	0.854344318	0.018031518	0.871255393	-3.393761527	DOWN
EPHX3	1.135386364	4.328534091	0.018290665	0.871255393	-3.400991529	UP
LGALS7B	1.485475	3.197776136	0.019296732	0.871255393	-3.428098943	UP
GPRIN2	1.066934091	7.496148864	0.019494878	0.871255393	-3.433266814	UP
GRPR	-1.098163636	2.637829545	0.019544553	0.871255393	-3.434553952	DOWN
IL22RA2	1.103836364	4.193120455	0.019703927	0.871255393	-3.43866099	UP
MMP20	1.101756818	2.007939773	0.019815792	0.871255393	-3.441523465	UP
DSG3	1.971315909	7.435539773	0.020521254	0.871255393	-3.459201995	UP
PGLYRP4	1.069077273	2.501261364	0.020869733	0.871255393	-3.467706008	UP
FAM19A3	1.074159091	4.630854545	0.020958951	0.871255393	-3.469859789	UP
KRT14	1.806229545	10.11669432	0.021128337	0.871255393	-3.47392311	UP
GABRA5	1.332684091	2.376546591	0.021526528	0.871255393	-3.483344941	UP

DLGAP1	1.237915909	5.048171591	0.0221225	0.871255393	-3.49711735	UP
TRIM29	1.08255	10.52544545	0.022292855	0.871255393	-3.500984146	UP
CD1A	1.272227273	3.9551	0.022609637	0.871950746	-3.508094543	UP
KRTDAP	1.321338636	1.7154375	0.023183326	0.874442356	-3.520714598	UP
GJB6	1.262638636	4.121401136	0.023355842	0.874442356	-3.524446887	UP
CES3	-1.110447727	5.288721591	0.023624283	0.874442356	-3.530198473	DOWN
LRRC31	-1.057911364	1.378382955	0.023670005	0.874442356	-3.531171409	DOWN
FOXG1	1.254393182	2.058571591	0.023960538	0.874442356	-3.537308924	UP
MMP7	1.297611364	11.27378977	0.024225592	0.874442356	-3.542841785	UP
SPRR1A	1.124043182	1.625628409	0.02475229	0.874442356	-3.553653819	UP
ABCC11	-1.619343182	3.704419318	0.025202337	0.874442356	-3.562706343	DOWN
SMR3B	-1.85825	1.888788636	0.026632916	0.874442356	-3.59041378	DOWN
MSLN	1.908618182	6.410425	0.026785646	0.874442356	-3.593280793	UP
SERHL2	-1.0408	5.672118182	0.026869349	0.874442356	-3.5948449	DOWN
SLURP1	1.097763636	2.840504545	0.027119064	0.874442356	-3.599481498	UP
ART3	1.546072727	5.905997727	0.027368451	0.874442356	-3.604068258	UP
SERPINB7	1.333338636	4.115380682	0.027674949	0.874442356	-3.609646748	UP
CALB2	1.326988636	7.937373864	0.028825824	0.874442356	-3.630038867	UP
MUC13	-1.007618182	2.265629545	0.030596281	0.874442356	-3.659822724	DOWN
DMRTA1	1.010472727	4.107334091	0.031243207	0.875065538	-3.670263447	UP
SERPINB2	1.321975	4.151089773	0.0315144	0.87627409	-3.674573862	UP
PLA2G4F	-1.03295	3.746259091	0.03217292	0.882176347	-3.684882831	DOWN

DDX43	-1.154940909	2.471670455	0.032184612	0.882176347	-3.685063887	DOWN
GPR87	1.146354545	4.466	0.033890549	0.900094402	-3.710776269	UP
DPP6	1.053061364	2.063430682	0.034392097	0.903526215	-3.718081089	UP
BRSK2	1.039018182	3.518684091	0.035502896	0.903526215	-3.733873625	UP
EDN2	1.066113636	6.379329545	0.036596076	0.903526215	-3.748922805	UP
TFF2	-1.114506818	1.849407955	0.03725016	0.906531755	-3.757705601	DOWN
EN1	1.174686364	8.521913636	0.037306773	0.906531755	-3.75845823	UP
COMP	1.085122727	8.887134091	0.037737344	0.906531755	-3.764143835	UP
COL10A1	1.016479545	9.046071591	0.038644242	0.906531755	-3.77590177	UP
KLK7	1.562297727	7.021264773	0.038737034	0.906531755	-3.777088602	UP
PAX7	-1.055759091	1.246145455	0.03878208	0.906531755	-3.777663686	DOWN
KRT6A	1.740440909	7.003172727	0.038911074	0.906531755	-3.779306666	UP
HTR3A	1.092806818	2.430144318	0.038912082	0.906531755	-3.779319492	UP
KRT4	1.482054545	3.526802273	0.039926044	0.909172932	-3.792039809	UP
SBSN	1.423422727	4.800788636	0.041666966	0.91451228	-3.813114909	UP
EMID2	1.133472727	3.890252273	0.042620786	0.91451228	-3.824275847	UP
ROPN1	1.122809091	7.006302273	0.043561063	0.91451228	-3.835026201	UP
SPRR3	1.039302273	1.484257955	0.044491345	0.91451228	-3.845426534	UP
FOXJ1	-1.177609091	2.818754545	0.044858308	0.914678361	-3.849466787	DOWN
ZIC4	1.014002273	4.211930682	0.045228612	0.91665872	-3.853508965	UP
CHRM1	1.025502273	6.182096591	0.046361747	0.921776029	-3.865666576	UP
KRT6B	1.376202273	10.39483068	0.046784579	0.921776029	-3.870123803	UP

PCDHA1	-1.077468182	4.006695455	0.047539157	0.921902079	-3.877974372	DOWN
MMP10	1.089386364	3.798256818	0.04844006	0.922005544	-3.887178246	UP
ZG16B	-1.291268182	7.595072727	0.048740062	0.922005544	-3.890203412	DOWN
